# Supplementary material for: The influences of urbanization on breeding behavior of American bullfrog (Aquarana catesbeiana) in South Korea
Source: PLoS One. 2025 Jun 17;20(6):e0326201. doi: 10.1371/journal.pone.0326201 (PMC12173361; doi:10.1371/journal.pone.0326201)
Supplement: S1 Fig — (A) air temperature, (B) humidity, (C) water temperature, (D) rainfall, and (E) wind speed. The data represents the daily average in each recording period. (PDF) [file pone.0326201.s001.pdf]

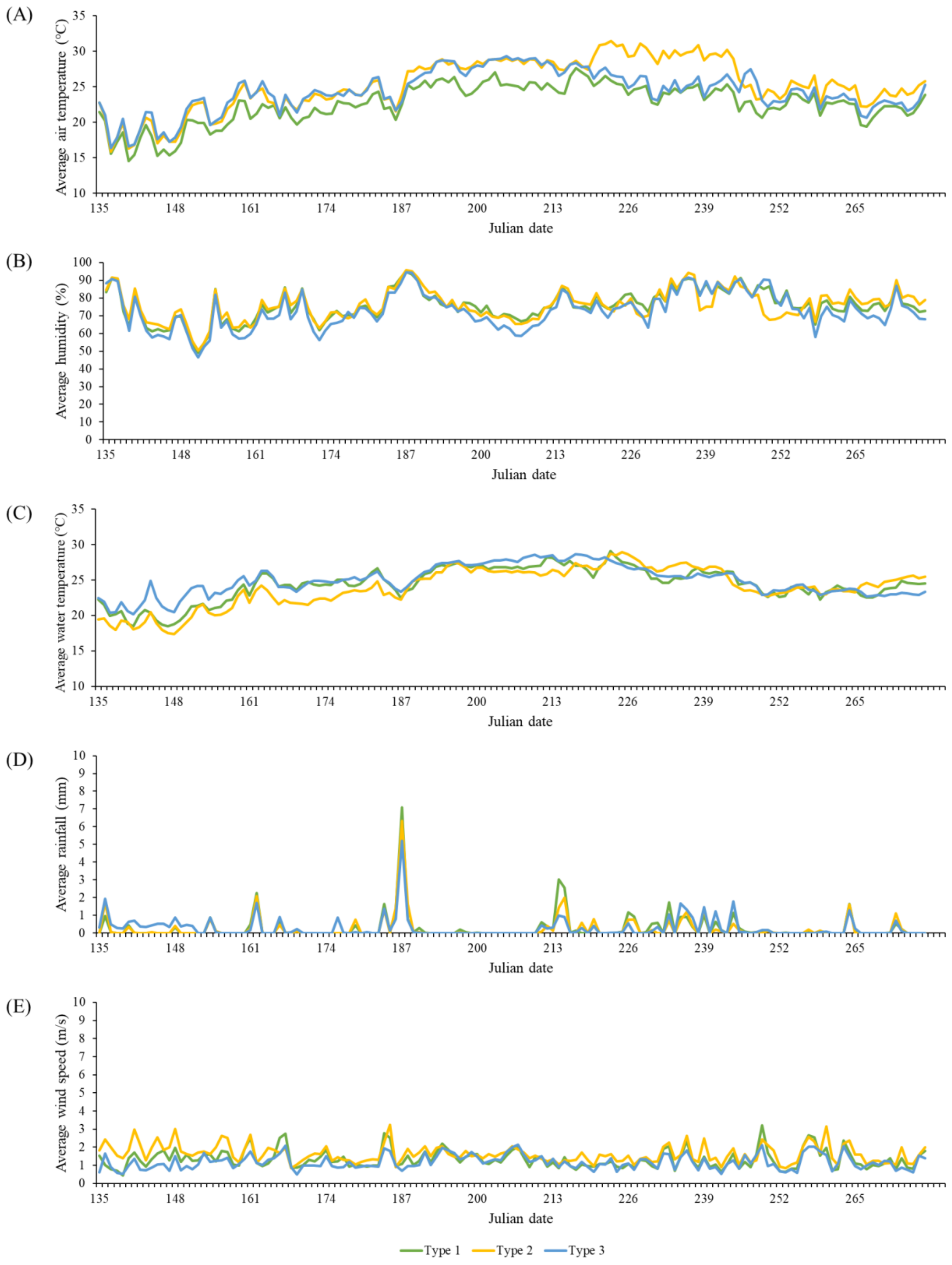

S1 Fig. Environmental variables at the study sites, categorized by their levels (Type). (A) air temperature, (B) humidity, (C) water temperature, (D) rainfall, and (E) wind speed. The data represents the daily average in each recording period.
