# Supplementary material for: The influences of urbanization on breeding behavior of American bullfrog (Aquarana catesbeiana) in South Korea
Source: PLoS One. 2025 Jun 17;20(6):e0326201. doi: 10.1371/journal.pone.0326201 (PMC12173361; doi:10.1371/journal.pone.0326201)
Supplement: S1 Table — (PDF) [file pone.0326201.s002.pdf]

S1 Table. Recording periods in each site.

No	Site	First recording	First call	Last call	Final recording
1	Dongnim	May 15th	May 20th	Oct 4th	Oct 5th
		16:00	16:00	2:00	18:00
2	Unpyeong	May 15th	May 23rd	Sep 5th	Oct 5th
		16:00	2:00	4:00	17:00
3	Bokman	Apr 26th	Apr 29th	Oct 3rd	Oct 5th
		21:00	19:00	14:00	18:00
4	Songhyeon	Apr 27th	Apr 27th	Oct 2nd	Oct 6th
		15:00	23:00	6:00	11:00
5	Ogang	Apr 27th	Apr 27th	Sep 21st	Oct 6th
		13:00	16:00	4:00	12:00
6	Naedong	May 15th	May 17th	Sep 18th	Oct 6th
		15:00	20:00	19:00	10:00
7	Useok	May 15th	May 15th	Aug 31st	Oct 6th
		13:00	22:00	6:00	12:00
8	Daeho	Apr 27th	May 19th	Aug 31st	Oct 6th
		12:00	1:00	23:00	11:00
9	Hansaebong	Apr 26th	Apr 26th	Aug 11th	Oct 5th
		12:00	12:00	4:00	15:00
10	Yangsan	May 15th	May 15th	Jun 13th	Oct 5th
		18:00	21:00	2:00	16:00
11	Dochon	May 15th	May 15th	-[#]	-
		0:00	0:00		
12	Suwan	May 15th	May 16th	Aug 9th	Oct 5th
		17:00	1:00	6:00	16:00

[#]At site no.11, recording during the later recording periods failed because the SM4 that was installed had malfunctioned. This site was excluded from the analysis.
