# Supplementary material for: The influences of urbanization on breeding behavior of American bullfrog (Aquarana catesbeiana) in South Korea
Source: PLoS One. 2025 Jun 17;20(6):e0326201. doi: 10.1371/journal.pone.0326201 (PMC12173361; doi:10.1371/journal.pone.0326201)
Supplement: S2 Table — The data are means±SD (min–max). Temperature is in °C, humidity in %, rainfall in mm, and wind speed in m/s. (PDF) [file pone.0326201.s003.pdf]

S2 Table. Environmental variables in the study sites categorized by different urbanization levels (site type). The data are means±SD (min–max). Temperature is in ℃, humidity in %, rainfall in mm, and wind speed in m/s.

Site	No. of minutes	Air temperature	Humidity	Water temperature	Rainfall	Wind speed
Type 1	10203	22.84±4.93 (10.3–36.9)	75.39±17.57 (23.4–98.3)	24.56±3.22 (16–33.5)	0.33±2.08 (0–40)	1.33±1.11 (0–7)
Type 2	9579	24.44±5.22 (10.3–37)	75.94±16.90 (23.7–99.9)	24.94±3.51 (16–33.4)	0.28±1.72 (0–34.5)	1.65±1.11 (0–8.4)
Type 3	5762	23.27±4.97 (11.3–37)	70.20±19.17 (22.4–98.4)	24.71±3.38 (16–33.5)	0.29±1.35 (0–24)	1.18±0.90 (0–12)
