# Supplementary material for: The influences of urbanization on breeding behavior of American bullfrog (Aquarana catesbeiana) in South Korea
Source: PLoS One. 2025 Jun 17;20(6):e0326201. doi: 10.1371/journal.pone.0326201 (PMC12173361; doi:10.1371/journal.pone.0326201)
Supplement: S3 Table — The numbers shown are Spearman correlation coefficients. (PDF) [file pone.0326201.s004.pdf]

S3 Table. The relationships between the 14 environmental variables and level of urbanization. The numbers shown are Spearman correlation coefficients.

Variables	JD	T	ST	AT	H	R	WS	WT	PC	DC	CN	DN	HN
Between 14 variables													
T	−0.004												
ST	−0.000	0.166**											
AT	0.507**	0.285**	0.398**										
H	0.242**	−0.349**	−0.366**	−0.368**									
R	−0.038**	0.007	0.017**	−0.130**	0.259**								
WS	−0.048**	0.282**	0.449**	0.375**	−0.374**	−0.007							
WT	0.635**	0.185**	0.100**	0.638**	−0.149**	−0.145**	0.153**						
PC	−0.323**	−0.109**	−0.257**	−0.324**	0.103**	0.001	−0.139**	−0.340**					
DC	−0.231**	−0.016**	−0.326**	−0.274**	0.081**	−0.027**	−0.106**	−0.277**	0.508**				
CN	−0.068**	0.040**	0.089**	0.080**	−0.138**	−0.025**	0.038**	0.040**	−0.017**	−0.058**			
DN	−0.146**	0.056**	0.054**	0.018**	−0.137**	0.012*	−0.016*	0.004	0.012*	−0.064**	0.074**		
HN	−0.158**	0.140**	0.040**	0.068**	−0.155**	−0.021**	0.024**	0.065**	−0.006	−0.043**	0.040**	0.282**	
Between 14 variables and urban degree													
Degree of urban	−0.196**	−0.003	−0.005	0.044**	−0.156**	−0.061**	0.0007	0.046**	0.017*	−0.062**	0.168**	0.295**	0.351**

*, $p < 0.05$; **, $p < 0.01$

AT: air temperature; CN: continuous traffic noise; DC: *Dryophytes japonica* calling index; DN: discontinuous traffic noise; H: humidity; HN: human noise; JD: Julian date; PC: *Pelophylax nigromaculatus* calling index; R: rainfall; ST: sunset and sunrise times; T: time; WS: wind speed; WT: water temperature.
