# Supplementary material for: The influences of urbanization on breeding behavior of American bullfrog (Aquarana catesbeiana) in South Korea
Source: PLoS One. 2025 Jun 17;20(6):e0326201. doi: 10.1371/journal.pone.0326201 (PMC12173361; doi:10.1371/journal.pone.0326201)
Supplement: S4 Table — All models are ranked by AIC for their ability to explain the influence of the factors on bullfrog calling activity. (PDF) [file pone.0326201.s005.pdf]

**S4 Table. The full model combining all 14 variables and alternative models containing various combinations of the 14 variables.** All models are ranked by AIC for their ability to explain the influence of the factors on bullfrog calling activity.

| No | Model | Variable | k[1] | AIC[2] | Rank |
|---|---|---|---|---|---|
| 1 | Abiotic | CN, WT | 4 | 3,975.92 | 1 |
| 2 | Abiotic | DN, H | 4 | 12,019.387 | 8 |
| 3 | Abiotic | CN, WT, H | 5 | 31,850.296 | 15 |
| 4 | Abiotic | DN, H, WT | 5 | 32,035.669 | 16 |
| 5 | Abiotic, Biotic | R, DN, H, DC | 6 | 19,158.817 | 11 |
| 6 | Abiotic, Biotic, Time | DC, R, T, WS | 6 | 17,626.818 | 10 |
| 7 | Abiotic, Date and Time | WT, CN, ST | 5 | 5,583.933 | 2 |
| 8 | Anthropogenic, Date and Time | CN, T | 4 | 8,688.868 | 5 |
| 9 | Biotic, Date and Time | PC, T | 4 | 8,513.525 | 4 |
| 10 | Biotic, Environmental | PC, WT | 4 | 6,127.019 | 3 |
| 11 | Biotic, Environmental | DC, WT, R, WS | 6 | 22,368.798 | 12 |
| 12 | Biotic, Environmental | AT, H, WT, PC, DC | 7 | 31,121.925 | 14 |
| 13 | Environmental | AT, R | 4 | 8,966.404 | 6 |
| 14 | Environmental | H, R | 4 | 12,228.254 | 9 |
| 15 | Environmental | AT, H | 4 | 43,238.195 | 18 |
| 16 | Environmental, Date and Time | R, T | 4 | 10,306.303 | 7 |
| 17 | Environmental, Date and Time | H, R, T | 5 | 35,349.322 | 17 |
| 18 | Environmental, Date and Time | H, T, WS | 5 | 44,348.519 | 19 |
| 19 | Global | AT, CN, DC, H, JD, R, T, WS, WT | 11 | 29,823.436 | 13 |

[1]Number of estimate parameter
[2]Akaike's information criterion (AIC)
AT: air temperature; CN: continuous traffic noise; DC: *Dryophytes japonica* Calling index; DN: discontinuous traffic noise; H: humidity; HN: human noise; JD: Julian date; PC: *Pelophylax nigromaculatus* calling index; R: rainfall; ST: sunset and sunrise times; T: time; WS: wind speed; WT: water temperature.
