# Supplementary material for: The influences of urbanization on breeding behavior of American bullfrog (Aquarana catesbeiana) in South Korea
Source: PLoS One. 2025 Jun 17;20(6):e0326201. doi: 10.1371/journal.pone.0326201 (PMC12173361; doi:10.1371/journal.pone.0326201)
Supplement: Attachment — Submitted filename: PONE-D-24-38252-RV.pdf [file pone.0326201.s006.pdf]

PLOS ONE

Urbanization influences breeding behavior in anurans: calling patterns of invasive American bullfrog (Aquarana catesbeiana) in areas with different levels of urbanization in South Korea

--Manuscript Draft--

**Manuscript Number:**	PONE-D-24-38252
**Article Type:**	Research Article
**Full Title:**	Urbanization influences breeding behavior in anurans: calling patterns of invasive American bullfrog (Aquarana catesbeiana) in areas with different levels of urbanization in South Korea
**Short Title:**	Calling patterns of bullfrog in urban areas
**Corresponding Author:**	Ha-Cheol Sung<br>Chonnam National University<br>Gwangju, N/A KOREA, REPUBLIC OF
**Keywords:**	urbanization; adaptation; anthropogenic noise; invasive species; calling activity; automated recording systems
**Abstract:**	Urbanization is often characterized by high levels of anthropogenic noise, which can mask or interfere with animal communication signals, especially those that use sound to communicate, making it challenging for individuals to detect and interpret acoustic cues. While calling is crucial for anuran breeding and communication, the coping mechanisms of city dwelling and cosmopolitan species in urbanized environments remain understudied. Given that invasive species have higher environmental adaptability than native species (because of habitat specificity and environmental sensitivity), we studied the calling patterns of the invasive American bullfrog (Aquarana catesbeiana) in different levels of urbanized areas in South Korea. We hypothesized that call patterns of A. catesbeiana could be influenced by both biotic and abiotic factors. We found an early onset of calling activities in urbanized A. catesbeiana populations, which suggests an urban-induced shift in breeding phenology. Additionally, urban populations show more intense diurnal calling activities but shorter breeding periods than non-urban populations. The results indicate water temperature and noise as the leading factors influencing calling activities in A. catesbeiana. Our study sheds light on the effect of urbanization on breeding activities and how an invasive species copes with modified environments in new areas. Our results will help future studies assessing the impacts of urbanization on biodiversity and animal behavior.
**Order of Authors:**	Ji-A Lee
	Md Mizanur Rahman, Ph.D.
	Seung-Ju Cheon
	Amaël Borzée, Ph.D.
	Ha-Cheol Sung
**Additional Information:**	
**Question**	**Response**
**Financial Disclosure**<br><br>Enter a financial disclosure statement that describes the sources of funding for the work included in this submission. Review the submission guidelines for detailed requirements. View published research articles from *PLOS ONE* for specific	Yes

examples.<br><br>This statement is required for submission and **will appear in the published article** if the submission is accepted. Please make sure it is accurate.<br><br>**Funded studies**<br>Enter a statement with the following details:<br>• Initials of the authors who received each award<br>• Grant numbers awarded to each author<br>• The full name of each funder<br>• URL of each funder website<br>• Did the sponsors or funders play any role in the study design, data collection and analysis, decision to publish, or preparation of the manuscript?<br><br>Did you receive funding for this work?	
Please add funding details.<br>as follow-up to "**Financial Disclosure**<br><br>Enter a financial disclosure statement that describes the sources of funding for the work included in this submission. Review the submission guidelines for detailed requirements. View published research articles from *PLOS ONE* for specific examples.<br><br>This statement is required for submission and **will appear in the published article** if the submission is accepted. Please make sure it is accurate.<br><br>**Funded studies**<br>Enter a statement with the following details:<br>• Initials of the authors who received each award<br>• Grant numbers awarded to each author<br>• The full name of each funder<br>• URL of each funder website<br>• Did the sponsors or funders play any role in the study design, data collection and analysis, decision to publish, or preparation of the manuscript?	This work was supported by the Korea Environment Industry & Technology Institute (KEITI) through the Project for the Development of Biological Diversity Threats Outbreak Management Technology (2018002270004), funded by the Korea Ministry of Environment (MOE).

Powered by Editorial Manager® and ProduXion Manager® from Aries Systems Corporation

Did you receive funding for this work?"	
Please select the country of your main research funder (please select carefully as in some cases this is used in fee calculation).<br>as follow-up to "**Financial Disclosure**<br><br>Enter a financial disclosure statement that describes the sources of funding for the work included in this submission. Review the submission guidelines for detailed requirements. View published research articles from *PLOS ONE* for specific examples.<br><br>This statement is required for submission and **will appear in the published article** if the submission is accepted. Please make sure it is accurate.<br><br>**Funded studies**<br>Enter a statement with the following details:<br>• Initials of the authors who received each award<br>• Grant numbers awarded to each author<br>• The full name of each funder<br>• URL of each funder website<br>• Did the sponsors or funders play any role in the study design, data collection and analysis, decision to publish, or preparation of the manuscript?<br><br>Did you receive funding for this work?"	KOREA, REPUBLIC OF - KR
**Competing Interests**<br><br>Use the instructions below to enter a competing interest statement for this submission. On behalf of all authors, disclose any competing interests that could be perceived to bias this work—acknowledging all financial support and any other relevant financial or non-financial competing interests.	The authors declare that the research was conducted in the absence of any commercial or financial relationships that could be construed as a potential conflict of interest.

Powered by Editorial Manager® and ProduXion Manager® from Aries Systems Corporation

This statement is required for submission and **will appear in the published article** if the submission is accepted. Please make sure it is accurate and that any funding sources listed in your Funding Information later in the submission form are also declared in your Financial Disclosure statement.<br><br>View published research articles from *PLOS ONE* for specific examples.<br><br>**NO authors have competing interests**<br><br>Enter: *The authors have declared that no competing interests exist.*<br><br>**Authors with competing interests**<br><br>Enter competing interest details beginning with this statement:<br><br>*I have read the journal's policy and the authors of this manuscript have the following competing interests: [insert competing interests here]*<br><br>* typeset	
**Ethics Statement**<br><br>Enter an ethics statement for this submission. This statement is required if the study involved:<br><br>• Human participants<br>• Human specimens or tissue<br>• Vertebrate animals or cephalopods<br>• Vertebrate embryos or tissues<br>• Field research<br><br>Write "N/A" if the submission does not require an ethics statement.<br><br>General guidance is provided below. Consult the submission guidelines for detailed instructions. **Make sure that all information entered here is included in the Methods section of the manuscript.**	Due to the non-invasive nature of this study, no ethical permission was required.

Powered by Editorial Manager® and ProduXion Manager® from Aries Systems Corporation

**Format for specific study types**<br><br>**Human Subject Research (involving human participants and/or tissue)**<br>• Give the name of the institutional review board or ethics committee that approved the study<br>• Include the approval number and/or a statement indicating approval of this research<br>• Indicate the form of consent obtained (written/oral) or the reason that consent was not obtained (e.g. the data were analyzed anonymously)<br><br>**Animal Research (involving vertebrate animals, embryos or tissues)**<br>• Provide the name of the Institutional Animal Care and Use Committee (IACUC) or other relevant ethics board that reviewed the study protocol, and indicate whether they approved this research or granted a formal waiver of ethical approval<br>• Include an approval number if one was obtained<br>• If the study involved *non-human primates*, add *additional details* about animal welfare and steps taken to ameliorate suffering<br>• If anesthesia, euthanasia, or any kind of animal sacrifice is part of the study, include briefly which substances and/or methods were applied<br><br>**Field Research**<br><br>Include the following details if this study involves the collection of plant, animal, or other materials from a natural setting:<br>• Field permit number<br>• Name of the institution or relevant body that granted permission	
**Data Availability**<br><br>Authors are required to make all data underlying the findings described fully available, without restriction, and from the time of publication. PLOS allows rare exceptions to address legal and ethical concerns. See the PLOS Data Policy and FAQ for detailed information.	Yes - all data are fully available without restriction

A Data Availability Statement describing where the data can be found is required at submission. Your answers to this question constitute the Data Availability Statement and **will be published in the article**, if accepted.<br><br>**Important:** Stating 'data available on request from the author' is not sufficient. If your data are only available upon request, select 'No' for the first question and explain your exceptional situation in the text box.<br><br>Do the authors confirm that all data underlying the findings described in their manuscript are fully available without restriction?	
**Describe where the data may be found in full sentences. If you are copying our sample text, replace any instances of XXX with the appropriate details.**<br><br>• If the data are **held or will be held in a public repository**, include URLs, accession numbers or DOIs. If this information will only be available after acceptance, indicate this by ticking the box below. For example: *All XXX files are available from the XXX database (accession number(s) XXX, XXX.*).<br>• If the data are all contained **within the manuscript and/or Supporting Information files**, enter the following: *All relevant data are within the manuscript and its Supporting Information files.*<br>• If neither of these applies but you are able to provide **details of access elsewhere**, with or without limitations, please do so. For example:<br><br>*Data cannot be shared publicly because of [XXX]. Data are available from the XXX Institutional Data Access / Ethics Committee (contact via XXX) for researchers who meet the criteria for access to confidential data.*<br><br>*The data underlying the results presented in the study are available*	All relevant data are within the paper or in the supplementary file.

from (include the name of the third party and contact information or URL).<br>• This text is appropriate if the data are owned by a third party and authors do not have permission to share the data.<br><br>* typeset	
Additional data availability information:	

Powered by Editorial Manager® and ProduXion Manager® from Aries Systems Corporation

Manuscript

Click here to access/download;Manuscript;Manuscript_Bullfrog calling patterns_MMR.docx

Urbanization influences breeding behavior in anurans: calling patterns of invasive American bullfrog (*Aquarana catesbeiana*) in areas with different levels of urbanization in South Korea

Ji-A Lee[1,†], Md Mizanur Rahman[2,†], Seung-Ju Cheon[1], Amaël Borzée[3], Ha-Cheol Sung[2*]

[1]Department of Biological Sciences and Biotechnology, Chonnam National University, Gwangju, South Korea

[2]Department of Biological Sciences, Chonnam National University, Gwangju, South Korea

[3]Laboratory of Animal Behaviour and Conservation, College of Life Sciences, Nanjing Forestry University, Nanjing, People's Republic of China

[†]These authors share first authorship

[*]Corresponding author: shcol2002@jnu.ac.kr

Abstract Urbanization is often characterized by high levels of anthropogenic noise, which can mask or interfere with animal communication signals, especially those that use sound to communicate, making it challenging for individuals to detect and interpret acoustic cues. While calling is crucial for anuran breeding and communication, the coping mechanisms of city dwelling and cosmopolitan species in urbanized environments remain understudied. Given that invasive species have higher environmental adaptability than native species (because of habitat specificity and environmental sensitivity), we studied the calling patterns of the invasive American bullfrog (*Aquarana catesbeiana*) in different levels of urbanized areas in South Korea. We hypothesized that call patterns of *A. catesbeiana* could be influenced by both biotic and abiotic factors. We found an early onset of calling activities in urbanized *A. catesbeiana* populations, which

suggests an urban-induced shift in breeding phenology. Additionally, urban populations show more intense diurnal calling activities but shorter breeding periods than non-urban populations. The results indicate water temperature and noise as the leading factors influencing calling activities in *A. catesbeiana*. Our study sheds light on the effect of urbanization on breeding activities and how an invasive species copes with modified environments in new areas. Our results will help future studies assessing the impacts of urbanization on biodiversity and animal behavior.

Keywords urbanization, adaptation, anthropogenic noise, invasive species, calling activity, automated recording systems

Introduction

Urban areas are landscapes where people live in high concentrations and use man-made structures, including their homes, workplaces, and transportation [1, 2]. Thus, they involve the conversion of natural and semi-natural habitats into urban infrastructure and human settlements. This process may influence the population status of many plant and animal species mainly due to habitat loss, habitat fragmentation/isolation, and degradation of habitat quality [3-6]. Furthermore, urban environments are often characterized by high levels of anthropogenic noise [7, 8], which can mask or interfere with animal communication signals, especially for animals that use sound to communicate, including amphibians, making it challenging for individuals to detect acoustic cues [9-13], and signals may be so garbled that they are unrecognizable or cannot contain any biologically significant information [14, 15]. Although a few species have developed strategies to mitigate the negative impacts of urban infrastructures and use them in enhancing call emission and propagation, eg., Mientien tree frog (*Kurixalus*

idiootocus) calls emit with longer duration, greater intensity in urban infrastructures [16], most of other amphibians do not show strong correlations between acoustic structure and the surrounding environment [17]. Thus, urban environments may cause a call emitter to lose fitness by failing to draw the attention of a mate or protect its territory and it brings a negative effect of reproduction and survival.

However, vocalization costs energy and may increase predation risk [18-20]. Hence, the calling activity of amphibians is periodic and can be affected by abiotic variables such as temperature and rainfall [21-23]. The abiotic environment is known to shift over time (daily or seasonally) [24, 25], yet human factors, including urbanization, can cause abrupt changes in the surrounding environment [26]. Among many other changes, the urban environment may cause high temperatures through heat islands [27]. It is already evident that many anuran species respond to rapid environmental changes, especially to temperature fluctuations [28-32, 21]. For example, *Allopa hazarensis* and *Nanorana vicina* were found to show quicker metamorphosis, smaller body size, increased frequency of developmental defects or edema, tail kinks, decreased fitness, and increased mortality at higher temperatures [33]. Moreover, temperature is reported to affect the number of males calling seasonally [19, 34].

Furthermore, surrounding noise in urban areas may induce higher energy costs and disrupt acoustic communications [34]. It is also reported that calling activities are generally performed in low-noise environments, whereas in response to noise, calls might be stopped or reduced in number [35-37]. In addition to the abiotic factors, biotic factors, such as the presence of predators and population density, may also influence frog calls [19, 38]. Thus, amphibians need to choose an appropriate active time when they can call

with less energy by minimizing influences of abiotic factors and biotic factors [39, 35-37].

Urbanization may also lead to the introduction of invasive species [40-42]. Together with creation of new habitats through urbanization, enhanced human movement and transportation have triggered the recent increase in the spread of invasive species [43]. One of the best examples of such spread of alien invasive species is the American bullfrog (*Aquarana catesbeiana*), which was introduced into more than 40 nations on four continents and is now one of the worst invasive amphibian species globally [44-46]. Like other invasive species, *A. catesbeiana* invasions might also be facilitated by their environmental adaptability [47, 46], which indicates that urban areas with changed environments may act as hotspots for their entry and settlement. However, the process by which they interact and cope with changes in urbanized areas, such as higher temperature and noise intensity, remained understudied [47]. Acoustic signaling is highly sensitive to various types of disturbances, making it potentially very important for studying animal behavior, can be examined in a wide variety of ecosystems, recorded, analyzed, synthesized, and played back with reasonable ease and at a low cost [48]. Furthermore, it is already evident that the calling behavior of anurans, unique and exclusive breeding behavior, can be influenced by habitat alteration and anthropogenic noise [49]. Considering the potentiality of using calls in studying anuran reproductive behavior, it may also help understand the impact of urbanization, a new ecosystem with shrunken breeding sites and changed environmental factors, on their breeding phenology [11].

Thus, we studied *A. catesbeiana* call patterns, number of calls over time, to understand their reproductive behavioral adaptations and breeding strategies in response to urbanization in South Korea to fill gaps in knowledge. We hypothesized that reduced

breeding sites, anthropogenic noise, and changed environmental factors in urbanized areas may shape call patterns of *A. catesbeiana* through increased competition, interruption by non-natural noise, raised temperature, and alteration in other factors. To test our hypothesis, we calculated the Urbanization Index of 12 potential breeding sites of *A. catesbeiana* and recorded their calls using an Automatic Recording System to observe the influence of urbanization intensity on call patterns.

Materials and Methods

Study species and site

The American bullfrog, *Aquarana catesbeiana,* previously *Lithobates catesbeiana*, belongs to the order Anura and family Ranidae (Fig 1a). They are considered one of the 100 world's worst invasive alien species and has dispersed over 40 countries (Fig 1b) [44]. Studies have reported an association between the American bullfrog and increased *Batrachochytrium dendrobatidis* (Bd) prevalence and population declines in many amphibian species [50-52]. Furthermore, a recent study showed that at least 84% of native anurans in the South Korea are threatened by *A. catesbeiana* [46]. Similar to other invaded countries, this species was brought from the US and Japan to the South Korea during the 1950s and 1970s, mostly to serve as a food source [53-55]. Now, *A. catesbeiana* has established local populations and can be observed all over the country and can be easily found in rivers and reservoirs [56, 57]. The breeding season of the species may last from June to August in the South Korea [56, 23, 46]. Male bullfrogs exhibit courtship behaviors, especially through calls, during the breeding season. They typically call for 0.6 to 1.5 seconds near the edge of their habitat, either alone or in choruses with several males [18, 58-61] .

After confirming the breeding activities of bullfrogs, we selected 12 study sites in Gwangju and Naju, in South Jeolla Province, South Korea (Fig 1c and 1d). The study sites were categorized according to the degree of urbanization, using Arcmap 10.8.1 (Esri, USA) and the Environmental Geographic Information Service (EGIS, https://egis.me.go.kr). The degree of urbanization was calculated using the percent of buildings in the land cover of a 1.6 km radius of each site [62, 63]. The area of 1.6 km radius was selected to cover the total home range of bullfrogs [46, 62]. We followed de Satgé et al. (2019)[63] method with a slight modification to apply it in Korea for measuring grades of urbanization and categorized the sites into three types: type 1, 0–3%; type 2, 5–10%; and type 3, 13% or above [63]. Four sites were selected for each type (Table 1). Type 1 included mostly rice paddies, fields, and mountains, whereas type 3 included mostly buildings (Fig 1d).

Data collection

We used Song Meter 4 (SM4, Wildlife Acoustic, USA) at a sample rate of 12 kHz and a resolution of 16 bits per sample. Recording was conducted for 162 days from April 26 to October 5, 2023, matching with the breeding period of the bullfrog in the South Korea [23, 46]. Initially, we surveyed and selected the reservoirs based on the previous reports on the presence of bullfrogs [57]. The recorders were installed after the confirmation of the presence of the bullfrogs at the study sites during the present surveys. Thus, there was a difference in the recording period from site to site. The earliest installation of the recorders was on 26 April 2021, whereas the latest installation of the recorders was on 15 May 2021 (S1A Table). The SM4 were installed about 1 m above ground level on the nearest tree from the reservoir edge. The distance of tree ranged from 0 to 1m. Considering the longer study period, we recorded first 5 minutes every hour for 24 hours

throughout the breeding season to make the study cost effective and unified. The SD cards and batteries were collected and replaced once a month. We used data from May to September for seasonal patterns and October to confirm the end of calling activity.

A total of 14 environmental, time, biotic, anthropogenic, and site variables were selected to identify the factors affecting the calling activity of bullfrogs [28, 30, 64]. Environmental variables included air temperature (AT), water temperature (WT), humidity (H), rainfall (R), and wind speed (WS). Temperature and humidity were recorded through a temperature and humidity data logger (RC-4HC, Elitech Technology, USA) installed at the same location as the SM4. Another data logger was installed at the water's edge at each site and measured the water temperature at a 15 cm depth. The data loggers were recorded every 30 minutes. Rainfall and wind speeds were based on data from the Korean Meteorological Administration (KMA, https://www.weather.go.kr), which was based on stations close to each site. Rainfall and wind speeds were recorded every hour. These recorded temperatures at a resolution of 0.1°C, humidity to 0.1 percentage point, rainfall to 0.1 mm, and wind speed to 0.1 m/s (S1B Table and S1 Fig).

Time variables included the Julian date (JD), time (T), and sunrise–sunset time (ST). The Julian date was recorded from 115 to 278 and time was recorded from 0 to 23. The Julian date was used to analyze the seasonal variation, and time for the daily variation in bullfrog calling activity. The sunrise–sunset time from the Korea Astronomy and Space Science Institute (KASI, https://astro.kasi.re.kr) was used to distinguish between day and night: 0 = from sunset to sunrise, night; 1 = from sunrise to sunset, day. As biotic variables, we included the most common species in Korea, the black-spotted pond frog (*Pelophylax nigromaculatus*, PC) and Japanese tree frog (*Dryophytes japonicus*, DC). The calls of these individuals were measured with the Calling Index (CI), as described in the next

subsection. The anthropogenic variables were traffic noise and human-presence noise. We counted the number of traffics and human noise to check the level of noise from passing vehicles and passing humans. The level of noise was increased with traffic volume and human presence [65]. The level of noise was measured by counting the noise events. All the recorded sounds (through SM4), a total of 25,544 minutes in 162 days, we analysis the 2nd - 3rd minute of recorded time and we analyzed to measure the noise in the study areas. The traffic noise was considered as "1" when car passed by [65, 66], while the intensity of noise was calculated as decibels below full scale (dBFS) using the Raven pro 1.6 (Cornell Lab of Ornithology, NY) between 90 Hz and 7000 Hz [67]. As zero dBFS represents maximum power, values for inband power are negative, with negative numbers closer to zero indicating more power [68]. We considered continuous noise (CN) if the aircraft and light vehicles passed by with high speed with a noise intensity of -46.79 ~ -3.23 dBFS, 90~5,668Hz, for more than 20 seconds. In such cases, we considered it as maximum "3". On the other hand, we considered discontinuous noise (DN) when vehicles passed by in a constant low speed, irresctive of types [69], with a noise intensity of -46.92 ~ -11.2 dBFS, 90~6124.3Hz, for less than 20 seconds. Human noise (HN) was considered as "1" when human voice and footsteps were heard within a 1-minute period and "0" when they were not. To avoid biases, a single person (the 1st author) heard and measured the noise level from the whole recordings. Traffic noise and human noise were counted using the recording file with Raven pro. Site (SI) variables was study sites, and this is the grades of urbanization from 1 to 12.

Call analysis

We used Raven Pro 1.6 to visualize calls and count them. To avoid biases and get the best quality data, the number of individuals was estimated by bullfrog advertisement calls over

2:00- to 3:00-minute periods in the recording file. The number of individuals was counted by measuring the Calling Index (CI). The CI was measured with values from 0 to 4: 0 = no individual called for 1 minute, 1 = one individual called for 1 minute, 2 = two individuals called for 1 minute, 3 = three individuals called for 1 minute, and 4 = four or more individuals' or indistinguishable calls for 1 minute [30]. Since anurans have different call characteristics that can be used in species [70], population [12] and individual identifications [71], each individual was identified through sonogram analysis. We recorded a total of 25,544 minutes in 162 days. After checking all recorded files, we analyzed a total of 7,436 minutes, excluding files where the sonogram could not be identified due to strong noise such as rain, or that did not contain any bullfrog calls. We identified 3,341 minutes in type 1, 2,186 minutes in type 2, and 1,909 minutes in type 3.

Statistics analysis

After confirming the von Mises distribution of our dataset ($p < 0.05$), the seasonal and daily calling activity of bullfrogs were analyzed through circular statistical analysis using Oriana 4 (Kovach Computing Services, Wales, UK). We calculated the mean vector (μ), mean vector length (r), median, concentration, circular variance, and circular standard deviation. To look for periodicity and irregularity in bullfrog call data, we used the Rayleigh test for uniformity. Furthermore, Watson–Williams *F* tests were conducted to compare the means and Mardia tests used for the comparison of significant distribution difference between two groups.

We used Generalized Linear Mixed Effects Models (GLMMs) to see the relationship between call activity and surrounding environmental, biotic, and abiotic factors and identify variables influencing the calling patterns of bullfrogs at the different study sites. The GLMMs were performed using a multinomial logistic regression because

the CI of the bullfrog, which is a dependent variable, is a discreet variable from 0 to 4. All variables except site were set as fixed effects and site was set as random effects Since there was no significant correlation (r > 0.8) and no multicollinearity between the 14 variables, all variables were used in the analysis (S1C Table). We created 19 models with a combination of 14 variables (S1D Table). Akaike's Information Criterion (AIC) was used to select the optimal model (S1D Table). We analyzed AIC and performed GLMMs using SPSS Statistics 23 (IBM Corp. Armonk, NY, USA).

Results

Seasonal pattern of calling activity

Our results revealed a significant seasonality throughout the entire recording period in all study sites (Rayleigh test $p < 0.05$; Table 2). We found an early onset of calling activities in urbanized *A. catesbeiana* populations, which suggests an urban-induced shift in breeding phenology. The highest CI output occurred in July at type 1 (lower urban sites) and in June at type 2 (medium urban sites) and type 3 (higher urban sites; Fig 2a). There were also differences in average active days between type 1 and type 3 (Watson–Williams test, $F = 16{,}476$, $p < 0.001$), and type 2 and type 3 (Watson–Williams test, $F = 6{,}316$, $p = 0.013$) sites. The number of active days was longest at type 1 sites and shortest for type 3. Additionally, we also found a difference in seasonal calling activity patterns between type 1 and type 3 (Mardia test, $W = 11.547$, $p = 0.003$). There is a difference in the seasonal distribution between type 1 and type 2, and type 1 and type 3. Type 1 had a decreasing pattern after peak in July, and type 3 had a decreasing pattern after peak in June.

Daily pattern of calling activity

Bullfrogs' calls were recorded all day and night, however, most calling activity was observed at night (20:00–06:00). The highest CI output was found at 03:00 at type 1 and type 2, and 02:00 at type 3 sites (Rayleigh test $p < 0.05$; Table 2; Fig 2b, c, d). The average activity times showed differences between type 1 and type 2 sites (Watson–Williams test $F = 7.61$, $p = 0.006$). The average activity time was at 01:28 in type 1 and 01:46 in type 2. The daily pattern of calling activity was significantly different between type 1 and type 3 (Mardia test $W = 23.126$, $p < 0.001$) and between type 2 and type 3 (Mardia test $W = 78.448$, $p < 0.001$) sites. There is a difference in the 24-hour activity distribution between type 1 and type 2, and type 1 and type 3. Calling activity peaked at around dawn in all types but was more widely distributed during the daytime at type 3 ~~sites~~.

Factors influencing calling activity

We checked the influence of 14 factors on the bullfrog calling index in the study sites. In the top five models, the abiotic model with continuous noise and water temperature had the lowest AIC (Table 3). Results of GLMM showed that there was more calling activity when the noise was less, and the water temperature was lower ($p < 0.001$; Table 4; Fig 3a, b). We found a decreased calling activity when the water temperature was more than 27℃ and less than 16℃. We recorded as few as 34 calls during continuous traffic noise (CN = 3), whereas, during no continuous traffic noise (CN = 0), the number of calls was as high as 7,430. This means the more traffic the less call activity. Also, our results suggest a negative correlation between CI and noise intensities (Fig 3c).

Discussion

The call patterns of the American bullfrog, a prolonged breeder, in natural and urban environments were observed in the current study. Bullfrogs have a long breeding season,

from April to August [23, 46]. However, we found them most active in June and July. The calling patterns were different among the three types of study areas categorized based on the grades of the urbanization.

Seasonal pattern of calling activity

We found sharp differences in seasonal activity, the onset of breeding season, and the calling behavior in *A. catesbeiana* between highly urbanized and non-urbanized areas. Although studies have reported June to August as the breeding season and calling activity period for bullfrogs [23, 46, 56], we recorded calls in late April to early October. However, we observed the onset of the calls in mid-May in most of the urbanized study sites (S1A Table). This might have resulted from the longer study period and continuous call recording used in our study and suggests a much longer breeding season for bullfrogs than the current knowledge posits. It also indicates that the areas with lower urbanization levels have a longer breeding period, running later into the season, and a later peak time. These differences in seasonal activity patterns, longer or shorter, might be attributed to the variations in the surrounding environmental factors of each habitat type [19]. For instance, more intense selection pressure and evolutionary conditions in urban environments due to artificial infrastructures, pollution, changes in abiotic factors, etc. may lead to behavioral modifications in urban dwelling species [72]. Changes in breeding phenology and duration may be such behavioral modifications, allowing bullfrogs in the urban environment to start breeding early and finish it quickly [73, 72, 5]. Previous studies have also found the breeding phenology of many amphibian species to be strongly dependent on the surrounding conditions [74-77].

Daily patterns of calling activity

In this study, calling activity of *A. catesbeiana* was mostly observed between 20:00 and 6:00. This finding is similar to those of previous reports, where researchers found nocturnal calling activities of *A. catesbeiana*, with the highest concentration of calls recorded at dawn [78]. Additionally, many other anurans, including other species of the genus *Aquarana*, also show nocturnal calling with the most activity at dawn [79, 80]. Generally, this might be a strategy to reduce the risk of dryness and dehydration [81, 82]. It might also be attributed to the lower temperature and less ambient noise during the night than in the daytime, which is conducive to efficient sound transmission [83].

However, the different types of study areas did not show significant differences in average daily activity periods but did in activity duration. Types 1 and 2 showed a similar amount of daily activity time, but type 3 showed a longer calling activity period. Additionally, some parts of the type 3 sites showed fierce population-level competition due to the narrowness of the reservoirs and the high numbers of individuals. Thus, there was a higher intensity of diurnal calls. It might be the strategy to avoid competition for breeding partners. Many authors have reported the formation of individuals forming a certain sphere of influence on the edge of the pond through breeding calls [59], a phenomenon of competition, and individuals attempting to avoid or reduce this competition by choosing temporal differences, even diurnal and nocturnal breeding activities [49, 84].

Factors influencing bullfrog calling activity

Continuous traffic noise and water temperature were the most influential factors affecting the bullfrog's calling activity in our study. We observed decreased calling activities on nights with more noise, especially, we found the lowest CIs in type 3, which had high levels of continuous noise. This might be an indication of a coping mechanism to deal

with anthropogenic and traffic noises in urban areas. Given that auditory masking by anthropogenic noise can reduce an individual's ability to detect voice, interfering with acoustic communications [85], the number of CIs and the participation of males in choruses can be negatively impacted [86, 87]. Reports on the impacts of urban noise on animals' acoustic communication [88, 89, 86], including the impact of traffic noise on the calling activities of anurans [37, 13], support our findings. In addition, the frequency ranges of traffic sounds and bullfrog calls also explain our results. The bullfrog's call has a frequency between 90 and 4000 Hz, which falls entirely within the frequency range of traffic noise, 50 to 7000 Hz; thus making it easily interrupted [90, 26]. It might also be a strategy to avoid excessive energy consumption by reducing calling activity during continuous traffic noise [91]. Being a cosmopolitan invader, *A. catesbeiana* seems to show high plasticity to cope with urban environments, arranging calling activities according to the intensity of ambient noise [92, 91].

The influence of temperature on anuran physiology and behavior—such as calling activity, especially in temperate monsoon climates—is also not surprising [93, 32, 94]. Previous observations on bullfrogs support our findings on the influence of temperature on their calling activity [95]. Our study suggests the temperature ranges for *A. catesbeiana* calling activity ranges from 16°C to 30°C. Researchers found most bullfrog calling activities in warm June, concluding that calling is likely limited by low temperatures in late autumn and early winter [96, 97]. Although studies indicate that bullfrogs are a summer (warmer weather) breeder, with water temperature portrayed as a potential predictor of calling activity, the lower threshold for water temperature was reported to vary [28, 98]. However, many previous studies have found results similar to ours [99, 28]. The differences in the findings might be attributed to the environmental conditions of the areas used in different studies. Thus, although a few studies have

suggested a minor impact of short-term weather, such as temperature, on prolonged breeders [30, 100], its influence is well-evident in bullfrogs' calling patterns in many others [101, 102].

Much research has reported on the relationship between temperature and the onset of breeding phenology in amphibians [103, 104]. Unlike other species, though *A. catesbeiana* depends on the seasonal temperature, it begins calling only when the water temperature reaches a suitable range [104]. During this study, type 3 sites (the most urbanized) showed a higher temperature than other types (S1 Fig), and thus, this may have influenced early breeding phenology and calling activities. Notably, previous studies have also found that many amphibian species start breeding earlier in urban areas than in rural areas, sometimes with longer breeding periods in rural areas [105, 106, 11]. Furthermore, urbanization causes variations in environmental factors, such as increasing temperatures through heat islands, that are crucial for amphibian calling activity and breeding phenology [19, 34, 27].

Thus, our study concluded that the bullfrog's calling activity is highly influenced by continuous traffic noise and environmental temperature. It also supports the existence of urbanization-induced patterns in the calling activities and breeding periods of amphibians. Although this study could not observe the subsequent impacts of shifts in breeding phenology, it sheds light on the effect of urbanization on breeding activities and how an invasive species copes with the changed environment in new areas. We recommend further intensive long-term research to better determine the onset of the breeding phenology and the extent to which the breeding period changes in different levels of urbanized and natural habitats and observe the related consequences. However, this study will help future researchers in studying the impacts of urbanization on

biodiversity and animal behavior. Additionally, our research provides information on the most active times for *A. catesbeiana* and when they are easily traceable (i.e., through calls) in all habitat types, which is important for identifying their population status and setting effective eradication measures in its invasive ranges [107-109].

Acknowledgments

The authors would like to thank everyone involved in the data collection and analysis.

Conflict of interest

The authors declare that the research was conducted in the absence of any commercial or financial relationships that could be construed as a potential conflict of interest.

Ethical statement

Due to the non-invasive nature of this study, no ethical permission was required.

Author contributions

Ji-A Lee: contributed to the study conception and design, sample collection, methodology, data collection and analysis, visualization, and writing- original draft, review, and editing. **Md Mizanur Rahman**: contributed to the study conception and design, critical feedback, supervision, and writing- original draft, review, and editing. **Seung-Ju Cheon**: contributed to the study conception and design, sample collection, visualization and review the draft. **Amaël Borzée**: contributed to the critical review. **Ha-Cheol Sung**: contributed to the conceptualization, supervision, funding acquisition, methodology, review the draft, and critical feedback. All authors read and approved the final draft.

Funding

This work was supported by the Korea Environment Industry & Technology Institute (KEITI) through the Project for the Development of Biological Diversity Threats Outbreak

Management Technology (2018002270004), funded by the Korea Ministry of Environment (MOE).

References

1. Pickett ST, Cadenasso ML, Grove JM. Boone CG, Groffman PM, Irwin E, et al. Urban ecological systems: Scientific foundations and a decade of progress. J. Environ. Manage. 2011; 92(3): 331–362. https://doi.org/10.1016/j.jenvman.2010.08.022

2. Wu J. Urban ecology and sustainability: The state-of-the-science and future directions. Landsc. Urban Plan. 2014; 125: 209–221. https://doi.org/10.1016/j.landurbplan.2014.01.018

3. Hamer AJ, McDonnell MJ. Amphibian ecology and conservation in the urbanising world: a review. Biol. Conserv. 2008; 141(10): 2432–2449. https://doi.org/10.1016/j.biocon.2008.07.020

4. Goddard MA, Dougill AJ, Benton TG. Scaling up from gardens: biodiversity conservation in urban environments. Trends Ecol. Evol. 2010; 25(2): 90–98. https://doi.org/10.1016/j.tree.2009.07.016

5. Liu Z, He C, Wu J. The relationship between habitat loss and fragmentation during urbanization: an empirical evaluation from 16 world cities. PLoS One. 2016; 11(4): e0154613. https://doi.org/10.1371/journal.pone.0154613

6. Wenzel A, Grass I, Raj V, Nölke N, Subramanya S, Tscharntke T. High losses of farmland birds and potential biocontrol along an urbanization gradient in a tropical megacity. Agric. Ecosyst. Environ. 2023; 354: 108571. https://doi.org/10.1016/j.agee.2023.108571

7. Slabbekoorn H, Peet M. Birds sing at a higher pitch in urban noise. Nature. 2003; 424(6946): 267–267. https://doi.org/10.1038/424267a

8. Higham V, Deal NDS, Chan YK, Chanin C, Davine E, Gibbings G, et al. Traffic noise drives an immediate increase in call pitch in an urban frog. J. Zool. 2021; 313(4): 307–315. https://doi.org/10.1111/jzo.12866

9. Brumm H. Animal communication: city birds have changed their tune. Curr. Biol. 2006;

16(23): R1003–1004. https://doi.org/10.1016/j.cub.2006.10.043

10. Parris KM. Anthropogenic noise constrains acoustic communication in urban-dwelling frogs. J. Acoust. Soc. Am. 2013; 19(1): 010055. https://doi.org/10.1121/1.4806379

11. Liu G, Kingsford RT, Callaghan CT, Rowley JJ. Anthropogenic habitat modification alters calling phenology of frogs. Glob. Chang. Biol. 2022; 28(21): 6194–6208. https://doi.org/10.1111/gcb.16367

12. Rahman MMR, Yun JY, Lee KH, Lee SH, Park SM, Ham CH, et al. Population-level call properties of endangered *Dryophytes suweonensissensu* lato (Anura: Amphibia) in South Korea. PeerJ. 2023; 11: e16492–e16492. https://doi.org/10.7717/peerj.16492

13. Luscier JD, Christopher A, Synan H, Wilson KB. Effects of light and noise pollution on occupancy of gray treefrogs (*Hyla versicolor*) and green frogs (*Lithobates clamitans*) in Syracuse, NY. Urban Ecosyst. 2023; 26(4): 941–953. https://dx.doi.org/10.1007/s11252-023-01329-9

14. Goutte S, Dubois A, Howard SD, Márquez R, Rowley JJL, Dehling JM, et al. How the environment shapes animal signals: A test of the acoustic adaptation hypothesis in frogs. J Evol Biol. 2018; 31(1):148-158. https://doi.org/10.1111/jeb.13210

15. Hardt B, Benedict L. Can you hear me now? A review of signal transmission and experimental evidence for the acoustic adaptation hypothesis. Bioacoustics. 2020; 30(6): 716–742. https://doi.org/10.1080/09524622.2020.1858448

16. Tan WH, Tsai CG, Lin C, Lin YK. Urban canyon effect: storm drains enhance call characteristics of the Mientien tree frog. J. Zool. 2014; 294(2): 77–84. https://doi.org/10.1111/jzo.12154

17. Gillard GL, Rowley JJL. Assessment of the acoustic adaptation hypothesis in frogs using large-scale citizen science data. J. Zool. 2023; 320(4): 271–281. https://doi.org/10.1111/jzo.13088

18. Ryan MJ, Tuttle MD, Taft LK. The costs and benefits of frog chorusing behavior. Behav. Ecol. Sociobiol. 1981; 8(4): 273–278. https://www.jstor.org/stable/4599395

19. Brooke PN, Alford RA, Schwarzkopf L. Environmental and social factors influence

chorusing behaviour in a tropical frog: examining various temporal and spatial scales. Behav. Ecol. Sociobiol. 2000; 49: 79–87. https://doi.org/10.1007/s002650000256

20. Kotiaho JS. Costs of sexual traits: a mismatch between theoretical considerations and empirical evidence. Biol. Rev. 2001; 76(3): 365–376. https://doi.org/10.1017/S1464793101005711

21. Schalk CM, Saenz D. Environmental drivers of anuran calling phenology in a seasonal Neotropical ecosystem. Austral. Ecol. 2016; 41(1): 16–27. https://doi.org/10.1111/aec.12281

22. Brodie S, Yasumiba K, Towsey M, Roe P, Schwarzkopf L. Acoustic monitoring reveals year-round calling by invasive toads in tropical Australia. Bioacoustics. 2021; 30(2): 125–141. https://doi.org/10.1080/09524622.2019.1705183

23. Groffen J, Andersen D, Borzée A. Breeding phenology and landscape use in all amphibian species from the Republic of Korea based on open-source data. Front. Environ. Sci. 2022; 10: 846481. https://doi.org/10.3389/fenvs.2022.846481

24. Slabbekoorn H, Ripmeester EAP. Birdsong and anthropogenic noise: implications and applications for conservation. Mol. Ecol. 2008; 17(1): 72–83. https://doi.org/10.1111/j.1365-294X.2007.03487.x

25. Kowarik I. Novel urban ecosystems, biodiversity, and conservation. Environ. Pollut. 2011; 159(8-9): 1974–1983. https://doi.org/10.1016/j.envpol.2011.02.022

26. Simmons AM, Narins PM. Effects of anthropogenic noise on amphibians and reptiles. In: Slabbekoorn H, Dooling R, Popper A, Fay R (ed) Effects of anthropogenic noise on animals. Springer; 2018. pp. 179–208.

27. Zhong S, Qian Y, Zhao C, Leung R, Wang H, Yang B, et al. Urbanization-induced urban heat island and aerosol effects on climate extremes in the Yangtze River Delta region of China. Atmos. Chem. Phys. 2017; 17(8): 5439–5457. https://doi.org/10.5194/acp-17-5439-2017

28. Oseen KL, Wassersug RJ. Environmental factors influencing calling in sympatric anurans. Oecologia. 2002; 133: 616–625. https://doi.org/10.1007/s00442-002-1067-5

29. Kopp K, Eterovick PC. Factors influencing spatial and temporal structure of frog assemblages at ponds in southeastern Brazil. J. Nat. Hist. 2006; 40(29-31): 1813–1830. https://doi.org/10.1080/00222930601017403

30. Saenz D, Fitzgerald LA, Baum KA, Conner RN. Abiotic correlates of anuran calling phenology: the importance of rain, temperature, and season. Herpetol. Monogr. 2006; 20(1): 64–82. https://doi.org/10.1655/0733-1347(2007)20[64:ACOACP]2.0.CO;2

31. Narins PM, Meenderink SW. Climate change and frog calls: long-term correlations along a tropical altitudinal gradient. Proc. R. Soc. B: Biol. Sci. 2014; 281(1783): 20140401. https://doi.org/10.1098/rspb.2014.0401

32. Rahman MMR. Impact of temperature fluctuations on gut histology of the Cricket frog, *Fejervarya limnocharis* (Anura: Dicroglossidae). J. Biosci. 2014; 22: 15–20. https://doi.org/10.3329/jbs.v22i0.30004

33. Saeed M, Rais M, Gray RJ, Ahmed W, Akram A, Gill S, et al. Rise in temperature causes decreased fitness and higher extinction risks in endemic frogs at high altitude forested wetlands in northern Pakistan. J. Therm. Biol. 2020; 95: 102809. https://doi.org/10.1016/j.jtherbio.2020.102809

34. McMahon TA, Rohr JR, Bernal XE. Light and noise pollution interact to disrupt interspecific interactions. Ecology. 2017; 98(5): 1290–1299. https://doi.org/10.1002/ecy.1770

35. Ryan MJ. Constraints and patterns in the evolution of anuran acoustic communication. In: Fritzsch B, The evolution of the amphibian auditory system. Wiley; 1988. pp. 637–677.

36. Sun JW, Narins PM. Anthropogenic sounds differentially affect amphibian call rate. Biol. Conserv. 2005; 121(3): 419–427. https://doi.org/10.1016/j.biocon.2004.05.017

37. Vargas-Salinas F, Cunnington GM, Amézquita A, Fahrig L. Does traffic noise alter calling time in frogs and toads? A case study of anurans in Eastern Ontario, Canada. Urban Ecosyst. 2014; 17: 945–953. https://doi.org/10.1007/s11252-014-0374-z

38. Hanna DE, Wilson DR, Blouin-Demers G, Mennill DJ. Spring peepers *Pseudacris crucifer* modify their call structure in response to noise. Curr. Zool. 2014; 60(4): 438–448. https://doi.org/10.1093/czoolo/60.4.438

39. Woolbright LL. Patterns of nocturnal movement and calling by the tropical frog *Eleutherodactylus coqui*. Herpetologica. 1985; 41: 1–9. https://www.jstor.org/stable/3892121

40. Towns DR, Daugherty CH. Patterns of range contractions and extinctions in the New Zealand herpetofauna following human colonisation. N. Z. J. Zool. 1994; 21(4): 325–339. https://doi.org/10.1080/03014223.1994.9518003

41. Kats LB, Ferrer RP. Alien predators and amphibian declines: review of two decades of science and the transition to conservation. Divers. Distrib. 2003; 9(2): 99–110. https://doi.org/10.1046/j.1472-4642.2003.00013.x

42. Shochat E, Lerman SB, Anderies JM, Warren PS, Faeth SH, Nilon CH. Invasion, competition, and biodiversity loss in urban ecosystems. BioScience. 2010; 60(3): 199–208. https://doi.org/10.1525/bio.2010.60.3.6

43. Bullock JM, Bonte D, Pufal G, da Silva C, Chapman DS, García C, et al. Human-mediated dispersal and the rewiring of spatial networks. Trends. Ecol. Evol. 2018; 33(12): 958–970. https://doi.org/10.1016/j.tree.2018.09.008

44. Lowe S, Browne M, Boudjelas S, De Poorter M. 100 of the world's worst invasive alien species: a selection from the global invasive species database (Vol. 12). Invasive Species Specialist Group; 2000. Pp. 6–7.

45. Ficetola GF, Thuiller W, Miaud C. Prediction and validation of the potential global distribution of a problematic alien invasive species—the American bullfrog. Divers. Distrib. 2007; 13(4): 476–485. https://doi.org/10.1111/j.1472-4642.2007.00377.x

46. Park HR, Rahman MM, Park SM, Choi JH, Kang HJ, Sung HC. Risk assessment for the native anurans from an alien invasive species, American bullfrogs (*Lithobates catesbeianus*), in South Korea. Sci. Rep. 2022; 12(1): 13143. https://doi.org/10.1038/s41598-022-17226-8

47. Borden JB, Flory SL. Urban evolution of invasive species. Front. Ecol. Environ. 2021; 19(3): 184–191. https://doi.org/10.1002/fee.2295.

48. Laiolo P. The emerging significance of bioacoustics in animal species conservation. Biol. Conserv. 2010; 143(7): 1635–1645. doi: 10.1016/j.biocon.2010.03.025

49. Cronin AD, Smit JA, Halfwerk W. Anthropogenic noise and light alter temporal but not spatial breeding behavior in a wild frog. Behav. Ecol. 2022; 33(6): 1115–1122. https://doi.org/10.1093/beheco/arac077

50. Miaud C, Dejean T, Savard K, Millery-Vigues A, Valentini A, Curt Grand Gaudin N, et al. Invasive North American bullfrogs transmit lethal fungus Batrachochytrium dendrobatidis infections to native amphibian host species. Biol. Invasions. 2016; 18(8): 2299–2308. https://doi.org/10.1007/s10530-016-1161-y

51. Borzée A, Kosch TA, Kim M, Jang Y. Introduced bullfrogs are associated with increased Batrachochytrium dendrobatidis prevalence and reduced occurrence of Korean treefrogs. PLoS ONE. 2017; 12(5): e0177860. https://doi.org/10.1371/0177860

52. Yap TA, Koo MS, Ambrose RF, Vreden-burg VT. Introduced Bullfrog facilitates pathogen invasion in the western United States. PLoS ONE. 2018; 13(4): e0188384. https://doi.org/10.1371/journal.pone.0188384

53. Oh HS, Hong CE. Current conditions of habitat for Rana catesbeiana and Trachemys scripta elegans imported to Jeju-do, including proposed management plans. Korean Journal of Environment and Ecology. 2007; 21(4): 311–317.

54. Park D, Min MS, Lasater K, Song JY, Suh JH, Son SH, et al. Conservation of amphibians in South Korea. In: Das MWI, Heatwole H (ed) Amphibian Biology, Conservation of Amphibians of the Eastern Hemisphere. Pelagic Publishing, Exeter; 2014. pp. 52–88.

55. Groffen J, Kong S, Jang Y, Borzée A. The invasive American bullfrog (*Lithobates catesbeianus*) in the Republic of Korea: history and recommendations for population control. Manag. Biol. Invasions. 2019; 10(3): 517–535. https://doi.org/2019.10.3.08

56. Lee JH, Jang HJ, Suh JH. Ecological guide book of herpetofauna in Korea. National Institute of Environmental Research; 2011. pp. 140–145.

57. Kang HJ, Koo KS, Sung HC. Current distribution of American bullfrog *Rana catesbeiana* Shaw, 1802 in the Republic of Korea. Bioinvasions Rec. 2019; 8(4): 942–946.

58. Capranica RR. The vocal repertoire of the bullfrog (*Rana catesbeiana*). Behaviour. 1968; 31(3-4): 302–324. https://doi.org/10.1163/156853968X00306

59. Emlen ST. Lek organization and mating strategies in the bullfrog. Behav. Ecol. Sociobiol. 1976; 1: 283–313. https://doi.org/10.1007/BF00300069

60. Laufer G, Gobel N, Martinez-Debat C, de Sá RO. Assessment of the calling detection probability throughout the day of two invasive populations of bullfrog (*Lithobates catesbeiana*) in Uruguay. Cuad. Herpetol. 2017; 31(1): 29–32. https://hdl.handle.net/20.500.12008/22630

61. Medeiros CI, Both C, Grant T, Hartz SM. Invasion of the acoustic niche: variable responses by native species to invasive American bullfrog calls. Biol. Invasions. 2017; 19: 675–690. https://doi.org/10.1007/s10530-016-1327-7

62. Ingram WM, Raney EC. Additional studies on the movement of tagged bullfrogs, *Rana catesbeiana* Shaw. Am. Midl. Nat. 1943; 29(1): 239–241. https://doi.org/10.2307/2420996

63. de Satgé J, Strubbe D, Elst J, De Laet J, Adriaensen F, Matthysen E. Urbanisation lowers great tit Parus major breeding success at multiple spatial scales. J. Avian. Biol. 2019; 50(11): 1–15. https://doi.org/10.1111/jav.02108.

64. Sommers MD, Randall LA, Barclay RM. Effects of environmental variables on the calling behaviour of Northern Leopard Frogs (*Lithobates pipiens*) in Alberta, Canada. Canadian Journal of Zoology. 2018; 96(2): 163-169. https://doi.org/10.1139/cjz-2016-0239

65. Parris KM, Schneider A. Impacts of traffic noise and traffic volume on birds of roadside habitats. Ecol. Soc. 2009; 14(1): 29. https://www.jstor.org/stable/26268029

66. Bautista LM, García JT, Calmaestra RG, Palacín C, Martín CA, Morales MB, et al. Effect of weekend road traffic on the use of space by raptors. Conserv. Biol. 2004; 18(3): 726-732. https://doi.org/10.1111/j.1523-1739.2004.00499.x

67. Rugg NM, Jenkins JM, Lesmeister DB. Western screech-owl occupancy in the face of an invasive predator. Glob. Ecol. Conserv. 2023; 48: e02753. https://doi.org/10.1016/j.gecco.2023.e02753

68. Cusano DA, Wiley D, Zeh JM, Kerr I, Pensarosa A, Zadra C, et al. Acoustic recording tags provide insight into the springtime acoustic behavior of sei whales in Massachusetts Bay. The Journal of the Acoustical Society of America. 2023; 154(6): 3543-3555. https://doi.org/10.1121/10.0022570

69. Morel J, Marquis-Favre C, Dubois D, Pierrette M. Road traffic in urban areas: A perceptual and cognitive typology of pass-by noises. Acta acustica united with Acustica. 2012; 98(1): 166-178. https://doi.org/10.3813/AAA.918502

70. Köhler J, Jansen M, Rodríguez A, Kok PJR, Toledo LF, Emmrich M., et al. (2017). The use of bioacoustics in anuran taxonomy: theory, terminology, methods and recommendations for best practice. Zootaxa. 2017; 4251(1): 1-124. https://doi.org/10.11646/zootaxa.4251.1.1

71. Pettitt BA, Bourne GR, Bee MA. Advertisement call variation in the golden rocket frog (*Anomaloglossus beebei*): evidence for individual distinctiveness. Ethology. 2013; 119(3): 244-256. https://doi.org/10.1111/eth.12058

72. Lowry H, Lill A, Wong BB. Behavioural responses of wildlife to urban environments. Biol. Rev. 2013; 88(3): 537–549. https://doi.org/10.1111/brv.12012

73. Kempenaers B, Borgström P, Loës P, Schlicht E, Valcu M. Artificial night lighting affects dawn song, extra-pair siring success, and lay date in songbirds. Curr. Biol. 2010; 20(19): 1735–1739. https://doi.org/10.1016/j.cub.2010.08.028

74. Ficetola GF, Maiorano L. Contrasting effects of temperature and precipitation change on amphibian phenology, abundance and performance. Oecologia. 2016; 181: 683–693. https://doi.org/10.1007/s00442-016-3610-9

75. Green DM. Amphibian breeding phenology trends under climate change: Predicting the past to forecast the future. Glob. Change. Biol. 2016; 23(2): 646–656. https://doi.org/10.1111/gcb.13390

76. Borzée A, Sin E, Oh S, Jang Y. Spring voices in Korean rice fields: the effect of abiotic variables and syntopic calls on the calling activity of the treefrog *Dryophytes suweonensis*. Asian Herp. Res. 2020; 11(4): 335–341. https://doi.org/10.16373/j.cnki.ahr.190046

77. Lenzi O, Grossenbacher K, Zumbach S, Lüscher B, Althaus S, Schmocker D, et al. Four decades of phenology in an alpine amphibian: trends, stasis, and climatic drivers. Peer Community J. 2023; 3: e15. https://doi.org/10.24072/pcjournal.240

78. Crouch WB, Paton PW. Assessing the use of call surveys to monitor breeding anurans in Rhode Island. J. Herpetol. 2002; 36(2): 185–192. https://doi.org/10.1670/0022-1511(2002)036[0185:ATUOCS]2.0.CO;2

79. Bridges AS, Dorcas ME. Temporal variation in anuran calling behavior: implications for surveys and monitoring programs. Copeia. 2000; 2000(2): 587–592. https://doi.org/10.1643/0045-8511(2000)000[0587:TVIACB]2.0.CO;2

80. Rocha CFD, Siqueira CC, Ariani CV, Vrcibradic D, Guedes DM, Kiefer MC, et al. Differential success in sampling of Atlantic Forest amphibians among different periods of the day. Braz. J. Biol. 2015; 75(2): 261–267. https://doi.org/10.1590/1519-6984.19412

81. Hartmann MT, Giasson LO, Hartmann PA, Haddad CF. Visual communication in Brazilian species of anurans from the Atlantic forest. J. Nat. Hist. 2005; 39(19): 1675–1685. https://doi.org/10.1080/00222930400008744

82. Laurie JV, Janalee PC. Herpetology; an introductory biology of amphibians and reptiles. ELSEVIER; 2013.

83. Gerhardt HC. The evolution of vocalization in frogs and toads. Annu. Rev. Ecol. Evol. Syst. 1994; 25: 293–324. https://doi.org/10.1146/annurev.es.25.110194.001453

84. Callaghan CT, Rowley JJ. A continental assessment of diurnality in frog calling behaviour. Austral. Ecol. 2021; 46(1): 65–71. https://doi.org/10.1111/aec.12959

85. Heinen-Kay JL, Kay AD, Zuk M. How urbanization affects sexual communication. Ecol. Evol. 2021; 11(24): 17625–17650. https://doi.org/10.1002/ece3.8328

86. Kaiser K, Scofield D, Alloush M, Jones R, Marczak S, Martineau K, et al. When sounds collide: the effect of anthropogenic noise on a breeding assemblage of frogs in Belize, Central America. Behaviour. 2011; 148(2): 215–232. https://doi.org/10.1163/000579510X551660

87. Zaffaroni-Caorsi V, Both C, Márquez R, Llusia D, Narins P, Debon M, et al. Effects of anthropogenic noise on anuran amphibians. Bioacoustics. 2023; 32(1): 90–120. https://doi.org/10.1080/09524622.2022.2070543

88. Reijnen R, Foppen R, Braak CT, Thissen J. The effects of car traffic on breeding bird populations in woodland. III. Reduction of density in relation to the proximity of main roads. J. Appl. Ecol. 1995; 32: 187–202. https://doi.org/10.2307/2404428

89. Pellet J, Guisan A, Perrin N. A concentric analysis of the impact of urbanization on the

threatened European tree frog in an agricultural landscape. Conserv. Biol. 2004; 18(6): 1599–1606. https://doi.org/10.1111/j.1523-1739.2004.0421a.x

90. Capranica RR. The evoked vocal response of the bullfrog-A study of communication by sound. The M.I.T. Press; 1965. p. 34.

91. Vargas-Salinas F, Amézquita A. Traffic noise correlates with calling time but not spatial distribution in the threatened poison frog *Andinobates bombetes*. Behaviour. 2013; 150(6): 569–584. https://doi.org/10.1163/1568539X-00003068

92. Rubbo MJ, Kiesecker JM. Amphibian breeding distribution in an urbanized landscape. Conserv. Biol. 2005; 19(2): 504–511. https://doi.org/10.1111/j.1523-1739.2005.000101.x

93. Blaustein AR, Belden LK, Olson DH, Green DM, Root TL, Kiesecker JM. Amphibian Breeding and Climate Change. Conserv. Biol. 2001; 15(6): 1804–1809. https://doi.org/10.1046/j.1523-1739.2001.00307.x

94. Forti LR, Hepp F, de Souza JM, Protazio A, Szabo JK. Climate drives anuran breeding phenology in a continental perspective as revealed by citizen-collected data. Divers. Distrib. 2022; 28: 2094–2109. https://doi.org/10.1111/ddi.13610

95. Kolson RA. Behavior of the bullfrog (*Rana catesbeiana*) with special emphasis on vocalization. Dissertation, The Ohio State University; 1973. pp. 69–133.

96. Kaefer ÍL, Boelter RA, Cechin SZ. Reproductive biology of the invasive bullfrog *Lithobates catesbeianus* in southern Brazil. Ann. Zool. Fenn. 2007; 44: 435–444. https://www.jstor.org/stable/23736845

97. Milne R, Bennett L, Hoyle M. Weather variability permitted within amphibian monitoring protocol and affects on calling Hylidae. Environmental monitoring and assessment. 2013; 185: 8879-8889. https://doi.org/10.1007/s10661-013-3221-4

98. Cook RP, Tupper TA, Paton PW, Timm BC. Effects of temperature and temporal factors on anuran detection probabilities at Cape Cod National Seashore, Massachusetts, USA: implications for long-term monitoring. Herpetol. Conserv. Biol. 2011; 6(2): 25–39.

99. Lillywhite HB. Behavioral temperature regulation in the bullfrog, *Rana catesbeiana*. Copeia. 1970; 1970(1):158–168. https://doi.org/10.2307/1441983

100. Steen DA, McClure CJ, Graham SP. Relative influence of weather and season on anuran calling activity. Can. J. Zool. 2013; 91(7): 462–467. https://doi.org/10.1139/cjz-2012-0266

101. Boatright-Horowitz SS, Cheney CA, Simmons AM. Atmospheric and underwater propagation of bullfrog vocalizations. Bioacoustics. 1999; 9(4): 257-280. https://doi.org/10.1080/09524622.1999.9753404

102. Govindarajulu P, Price WS, Anholt BR. Introduced bullfrogs (*Rana catesbeiana*) in western Canada: has their ecology diverged? J. Herpetol. 2006; 40(2): 249–260. https://doi.org/10.1670/68-05A.1

103. Duellman WE, Trueb L. Biology of amphibians. John Hopkins University Press; 1994. p. 13, p. 19.

104. Gibbs JP, Breisch AR. Climate warming and calling phenology of frogs near Ithaca, New York, 1900–1999. Conserv. Biol. 2001; 15(4): 1175–1178. https://doi.org/10.1046/j.1523-1739.2001.0150041175.x

105. Møller A, Díaz M, Grim T, Dvorská A, Flensted-Jensen E, Ibáñez-Álam, et al, Effects of urbanization on bird phenology: a continental study of paired urban and rural populations. Clim. Res. 2015; 66(3): 185–199. https://doi.org/10.3354/cr01344

106. Hart AG, Hesselberg T, Nesbit R, Goodenough AE. The spatial distribution and environmental triggers of ant mating flights: using citizen-science data to reveal national patterns. Ecography. 2018; 41(6): 877–888. https://doi.org/10.1111/ecog.03140

107. Lemckert F, Mahony M. Core calling periods of the frogs of temperate New South Wales, Australia. Herpetol. Conserv. Biol. 2008; 3(1): 71–76. https://hdl.handle.net/1959.13/39659

108. Measey GJ, Stevenson BC, Scott T, Altwegg R, Borchers DL. Counting chirps: acoustic monitoring of cryptic frogs. J. Appl. Ecol. 2016; 54(3): 894–902. https://doi.org/10.1111/1365-2664.12810

109. Shine R, Everitt C, Woods D, Pearson DJ. An evaluation of methods used to cull invasive cane toads in tropical Australia. J. Pest. Sci. 2018; 91(3): 1081–1091. https://doi.org/10.1007/s10340-018-0975-z

Table 1. Study sites at Gwangju and Naju in the South Korea, with their locations and degrees of urbanization

Group	Site	Degree of urbanization (%)	Latitude (N)	Longitude (E)
**Type 1**	Dongnim	0.76	35.2068	126.6995
	Unpyeong	0.88	35.1491	126.7290
	Bokman	1.70	35.1615	126.6590
	Songhyeon	2.07	34.9642	126.8020
**Type 2**	Ogang	5.83	35.0279	126.7620
	Naedong	6.47	35.0141	126.7090
	Useok	7.29	35.0247	126.7720
	Daeho	9.60	35.0410	126.7200
**Type 3**	Hansaebong	13.24	35.2123	126.8933
	Yangsan	19.47	35.2028	126.8721
	Dochon	21.23	35.2104	126.8236
	Suwan	23.80	35.1887	126.8209

Table 2. Circular statistical analysis of *Aquarana catesbeiana* calling activity in different types of study sites.

Variables	Type 1	Type 2	Type 3
Seasonal patterns			
**No. of observations**	124	108	87
**Mean vector (μ)**	190.58°	166.532°	159.159°
**Mean date (yy/mm/dd)**	21/07/13	21/06/18	21/06/11
**Length of mean vector (r)**	0.869	0.931	0.965
**Concentration**	4.111	7.491	14.699
**Circular variance (S)**	0.131	0.069	0.035
**Circular standard deviation (SD)**	30.411°	21.718°	15.213°
**Rayleigh test (Z)**	75.449	44.175	48.46
**Rayleigh test (*p*)**	< 0.0001	< 0.0001	< 0.0001
Daily patterns			
**No. of observations**	3523	2324	1927
**Mean vector (μ)**	22.042°	26.74°	24.961°
**Mean time (hh:mm)**	01:28	01:46	01:39
**Length of mean vector (r)**	0.709	0.679	0.646
**Median**	30°	30°	30°
**Concentration**	2.064	1.883	1.713
**Circular variance (S)**	0.291	0.321	0.354
**Circular standard deviation (SD)**	47.507°	50.43°	53.562°
**Rayleigh test (Z)**	4779.385	2293.623	1948.042
**Rayleigh test (*p*)**	< 0.0001	< 0.0001	< 0.0001

Table 3. Comparison of the top five alternative models ranked by AIC to explain the influence of factors on bullfrog calling activity.

No	Model	Variable	k[1]	AIC[2]	delta AIC[3]	w[4]
**1**	Abiotic	CN, WT	4	3,975.92	0	1
**2**	Abiotic, Date, and Time	WT, CN, ST	5	5,583.93	1,608.02	0
**3**	Biotic, Environmental	PC, WT	4	6,127.02	2,151.10	0
**4**	Biotic, Date, and Time	PC, T	4	8,513.53	4,537.61	0
**5**	Anthropogenic, Date, and Time	CN, T	4	8,688.87	4,712.95	0

[1]Number of estimate parameter
[2]Akaike's information criterion (AIC)
[3]Difference between the top model and given model
[4]Akaike weight
CN: continuous traffic noise; PC: *Pelophylax nigromaculatus* calling index; ST: sunset and sunrise times; T: time; WT: water temperature.

Table 4. Parameter estimates (coefficients and standard error) from the best-supported model for bullfrog calling activity.

Variable	*Coefficient*	*SE*	*t*	*p*
Bullfrog calling index 1				
**(intercept)**	−1.132	0.198	−5.716	< 0.001*
**CN**	−0.174	0.053	−3.264	0.001*
**WT**	−0.032	0.008	−4.084	<0.001*
Bullfrog calling index 2				
**(intercept)**	−0.557	0.232	−2.399	0.016*
**CN**	−0.450	0.087	−5.161	< 0.001*
**WT**	−0.072	0.009	−7.564	< 0.001*
Bullfrog calling index 3				
**(intercept)**	−0.211	0.257	−0.822	0.411
**CN**	−0.996	0.171	−5.843	< 0.001*
**WT**	−0.096	0.011	−9.009	< 0.001*
Bullfrog calling index 4				
**(intercept)**	1.303	0.177	7.382	< 0.001*
**CN**	−2.019	0.252	−7.998	< 0.001*
**WT**	−0.123	0.007	−16.762	< 0.001*

*= significant
CN: continuous traffic noise; WT: water temperature

Figure captions

Fig 1. Study species and sites. (a) Invasive American Bullfrog (*Aquarana catesbeiana*). (b) The worldwide distribution of *A. catesbeiana* populations. The data were retrieved from GBIF (https://www.gbif.org/). (c) Map of the South Korea showing the study areas. (d) Studied reservoirs in Gwangju and Naju-si with urbanization index represented by color (see side panel).

Fig 2. Seasonal and daily calling activity patterns of the bullfrog in different types of study sites. (a) The seasonal calling pattern. Circles represent habitat type 1 (blue), squares habitat type 2 (magenta), and triangles habitat type 3 (green). (b) The daily calling pattern in type 1 sites. (c) The daily calling pattern in type 2 sites. (d) The daily calling pattern in type 3 sites. The length of the sections indicates the calling index of bullfrogs in the corresponding time range. The red line depicts the mean vector length (r), which indicates the concentration of species' calling activity during the day.

Fig 3. Important factors affecting the bullfrog calling index in study sites. (a) The influence of continuous noise, which was included in the top-ranked GLMM predicting the calling index. (b) The influence of water temperature, which was also included in the top-ranked GLMMs. (c) The relationship between calling index and noise intensities.

Figure 1

Click here to access/download;Figure;Figure 1.jpg

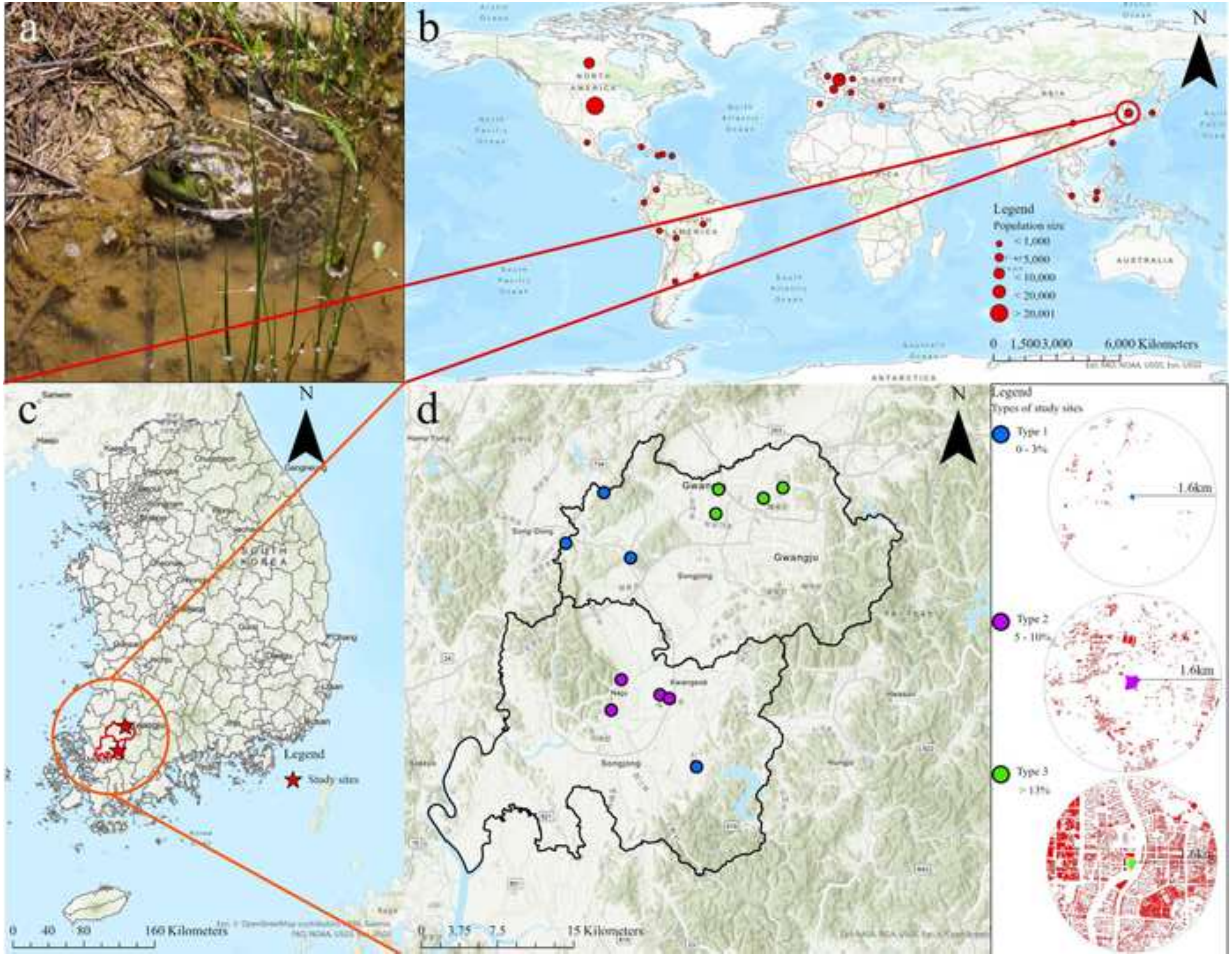

Figure 2

Click here to access/download;Figure;Figure 2.jpg

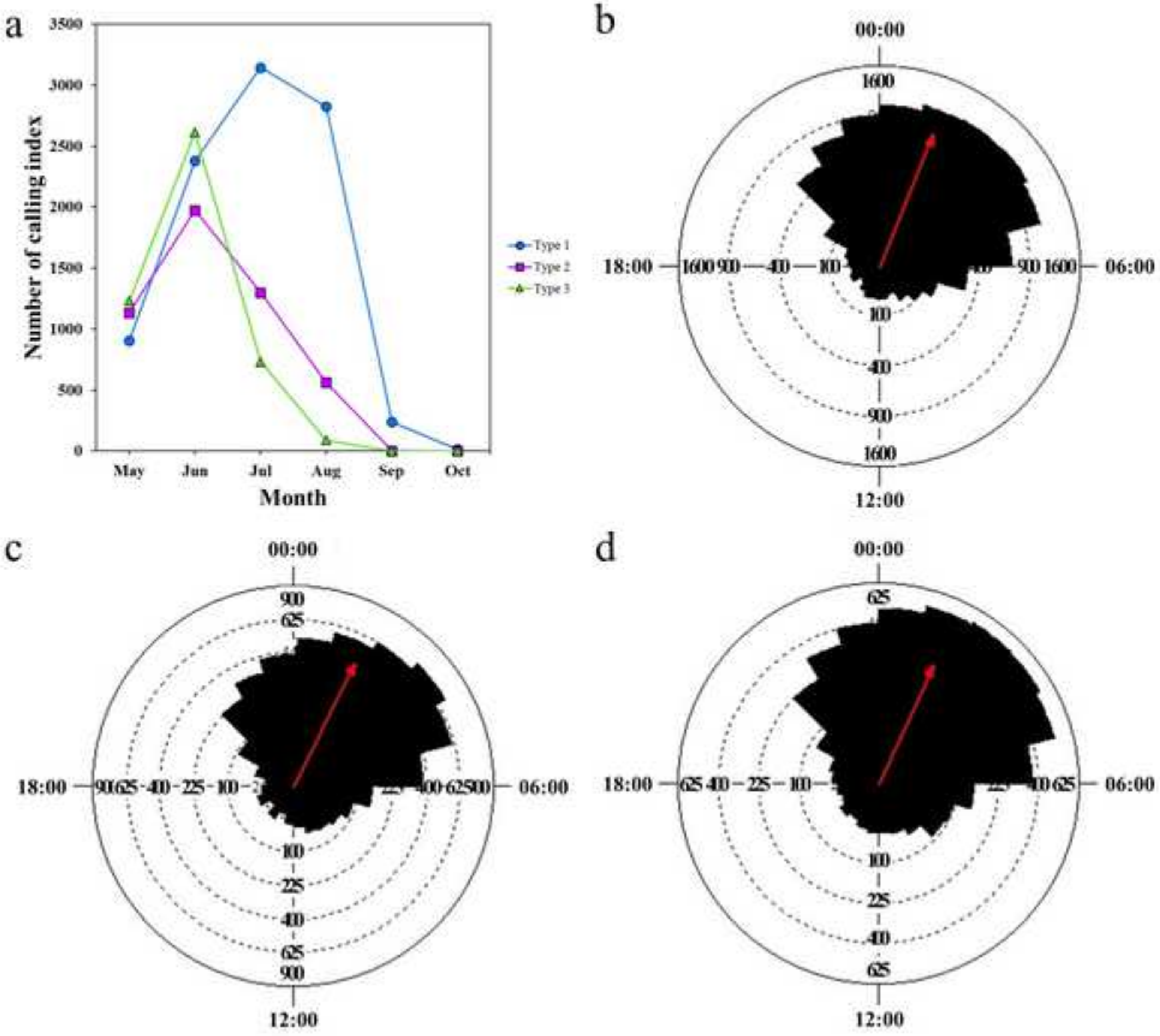

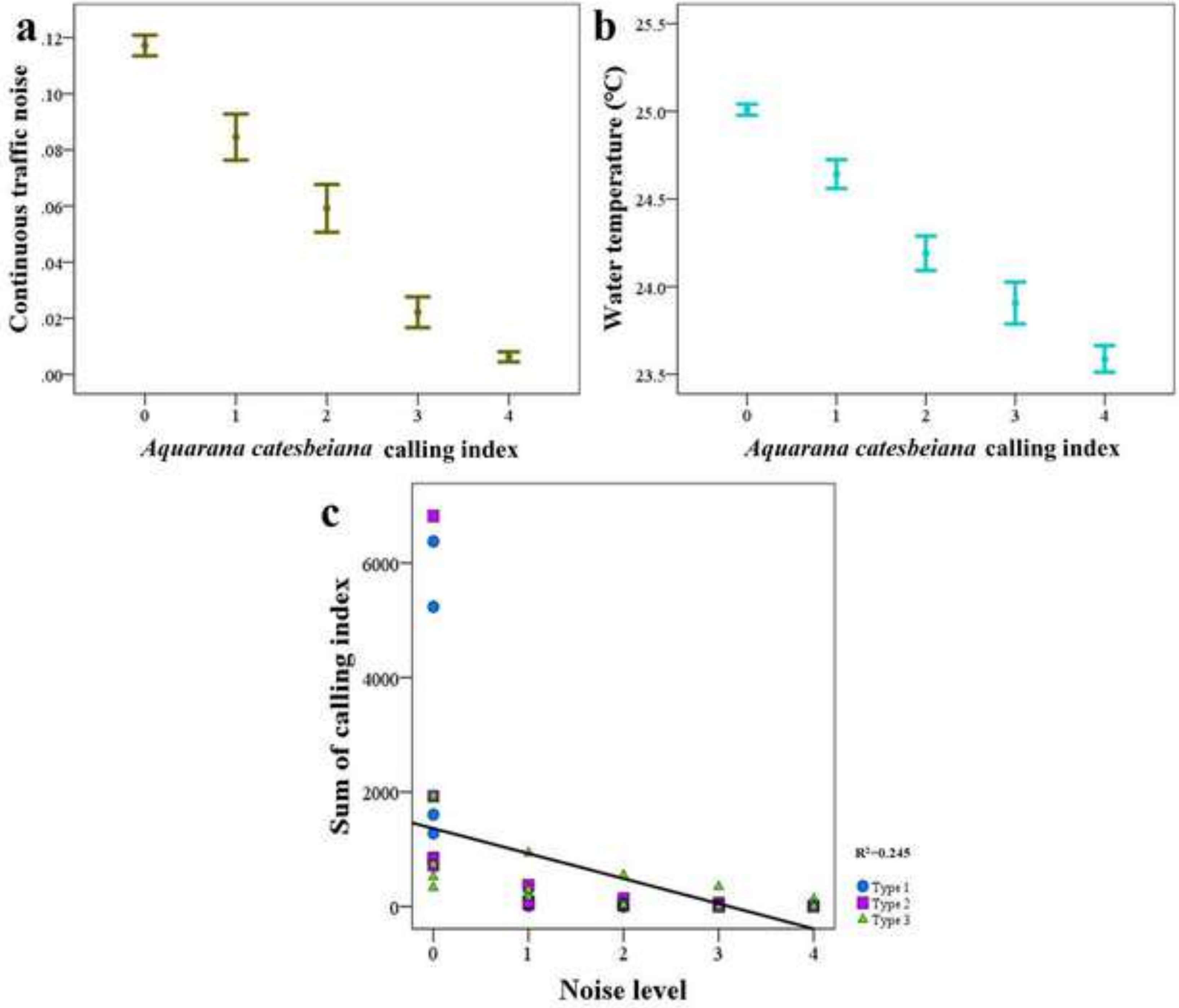
a
Continuous traffic noise
.12
.10
.08
.06
.04
.02
.00
0
1
2
3
4
Aquarana catesbeiana calling index
b
Water temperature (°C)
25.5
25.0
24.5
24.0
23.5
0
1
2
3
4
Aquarana catesbeiana calling index
c
Sum of calling index
6000
4000
2000
0
0
1
2
3
4
Noise level
R²=0.245
Type 1
Type 2
Type 3

Click here to access/download
Supporting Information
Supplimentary file_Bullfrog calling patterns_MMR.docx
