# Supplementary material for: The influences of urbanization on breeding behavior of American bullfrog (Aquarana catesbeiana) in South Korea
Source: PLoS One. 2025 Jun 17;20(6):e0326201. doi: 10.1371/journal.pone.0326201 (PMC12173361; doi:10.1371/journal.pone.0326201)
Supplement: Attachment — Submitted filename: Response to Reviewers_MMR.docx [file pone.0326201.s008.docx]

**Point-by-point Response to the Editor’s and Reviewers’ comments**

**Editor’s Comments:**

**Comment:**

After this first review round, both reviewers reached very positive views regarding your study. Therefore, I am recommending a minor review before the second (and possibly last) review round. Please consider all suggestions made by the reviewers to improve the next version of your manuscript.

***Response:***

We highly appreciate your efforts. We are glad that although the review process took a long time, it came up with very positive result. We hope next rounds of the publication of our manuscript will be faster and smoother. We thank you for your affirmative recommendation for our manuscript. We considered all suggestions made by the reviewers to improve the revised version of our manuscript. Herein, we are attaching a point-by-point response to the reviewers’ comments with details of the corrections and modifications we made to our manuscript. Please check and let us know if you have any additional comments. If necessary, we are open to revise our manuscript further to improve it to fulfill the high standard of your journal and editorial process.

**PLOS ONE**

**Journal Requirements:**

**Comment:**

1. Please ensure that your manuscript meets PLOS ONE's style requirements, including those for file naming. The PLOS ONE style templates can be found at

https://journals.plos.org/plosone/s/file?id=wjVg/PLOSOne_formatting_sample_main_body.pdf and 
https://journals.plos.org/plosone/s/file?id=ba62/PLOSOne_formatting_sample_title_authors_affiliations.pdf

***Response:***

Thanks for your comment. We have revised our manuscript accordingly.

**Comment:**

2. In your Methods section, please provide additional information regarding the permits you obtained for the work. Please ensure you have included the full name of the authority that approved the field site access and, if no permits were required, a brief statement explaining why.

***Response:***

We added it in lines 129 – 130.

**Comment:**

3. Thank you for stating the following financial disclosure:

“This work was supported by the Korea Environment Industry & Technology Institute (KEITI) through the Project for the Development of Biological Diversity Threats Outbreak Management Technology (2018002270004), funded by the Korea Ministry of Environment (MOE).”

Please state what role the funders took in the study.  If the funders had no role, please state: "The funders had no role in study design, data collection and analysis, decision to publish, or preparation of the manuscript." If this statement is not correct you must amend it as needed.

Please include this amended Role of Funder statement in your cover letter; we will change the online submission form on your behalf.

***Response:***

We highly appreciate your valuable advice. We have stated the role of the funders clearly and mentioned it in the cover letter as per your instructions.

**Comment:**

4. Please note that funding information should not appear in the Acknowledgments section or other areas of your manuscript. We will only publish funding information present in the Funding Statement section of the online submission form. Please remove any funding-related text from the manuscript.

***Response:***

Thanks for your comment. We have deleted the funding information from the manuscript.

**Comment:**

5. We note that Figure 1 in your submission contain map/satellite images which may be copyrighted. All PLOS content is published under the Creative Commons Attribution License (CC BY 4.0), which means that the manuscript, images, and Supporting Information files will be freely available online, and any third party is permitted to access, download, copy, distribute, and use these materials in any way, even commercially, with proper attribution. For these reasons, we cannot publish previously copyrighted maps or satellite images created using proprietary data, such as Google software (Google Maps, Street View, and Earth). For more information, see our copyright guidelines: http://journals.plos.org/plosone/s/licenses-and-copyright.
We require you to either (1) present written permission from the copyright holder to publish these figures specifically under the CC BY 4.0 license, or (2) remove the figures from your submission:
1) You may seek permission from the original copyright holder of Figure 1 to publish the content specifically under the CC BY 4.0 license.  
We recommend that you contact the original copyright holder with the Content Permission Form (http://journals.plos.org/plosone/s/file?id=7c09/content-permission-form.pdf) and the following text:
“I request permission for the open-access journal PLOS ONE to publish XXX under the Creative Commons Attribution License (CCAL) CC BY 4.0 (http://creativecommons.org/licenses/by/4.0/). Please be aware that this license allows unrestricted use and distribution, even commercially, by third parties. Please reply and provide explicit written permission to publish XXX under a CC BY license and complete the attached form.”
Please upload the completed Content Permission Form or other proof of granted permissions as an ""Other"" file with your submission.
In the figure caption of the copyrighted figure, please include the following text: “Reprinted from [ref] under a CC BY license, with permission from [name of publisher], original copyright [original copyright year].”
2) If you are unable to obtain permission from the original copyright holder to publish these figures under the CC BY 4.0 license or if the copyright holder’s requirements are incompatible with the CC BY 4.0 license, please either i) remove the figure or ii) supply a replacement figure that complies with the CC BY 4.0 license. Please check copyright information on all replacement figures and update the figure caption with source information. If applicable, please specify in the figure caption text when a figure is similar but not identical to the original image and is therefore for illustrative purposes only.

The following resources for replacing copyrighted map figures may be helpful:
USGS National Map Viewer (public domain): http://viewer.nationalmap.gov/viewer/
The Gateway to Astronaut Photography of Earth (public domain): http://eol.jsc.nasa.gov/sseop/clickmap/
Maps at the CIA (public domain): https://www.cia.gov/library/publications/the-world-factbook/index.html and https://www.cia.gov/library/publications/cia-maps-publications/index.html
NASA Earth Observatory (public domain): http://earthobservatory.nasa.gov/
Landsat: http://landsat.visibleearth.nasa.gov/
USGS EROS (Earth Resources Observatory and Science (EROS) Center) (public domain): http://eros.usgs.gov/#
Natural Earth (public domain): http://www.naturalearthdata.com/

***Response:***

We appreciate your concern. The maps were generated using Arcmap 10.8.1 (ESRI, USA; <https://support.esri.com/en/products/desktop/arcgis-desktop/arcmap/10-8-1>), which is free to use and now, we referred the source in the figure caption. Please, check lines 108 – 113 of the revised manuscript. We did the same thing in one of our recent papers published in PlosOne, <https://doi.org/10.1371/journal.pone.0281808.g001>.

**Comment:**

6. Please include captions for your Supporting Information files at the end of your manuscript, and update any in-text citations to match accordingly. Please see our Supporting Information guidelines for more information: http://journals.plos.org/plosone/s/supporting-information.

***Response:***

Thanks for your suggestion. We have included captions for our Supporting Information files at the end of our manuscript and updated in-text citations following your suggestion.

**Comment:**

7. Please review your reference list to ensure that it is complete and correct. If you have cited papers that have been retracted, please include the rationale for doing so in the manuscript text, or remove these references and replace them with relevant current references. Any changes to the reference list should be mentioned in the rebuttal letter that accompanies your revised manuscript. If you need to cite a retracted article, indicate the article’s retracted status in the References list and also include a citation and full reference for the retraction notice.

***Response:***

We appreciate your concern. We have checked and revised the reference list accordingly.

**Comments from Reviewer #1:**

**Comment:**

Reviewer #1: The aim of the study was to evaluate the impact of the urbanization on the calling activity pattern of an invasive frog species. Overall, the study is well designed and provides data to respond to the proposed objectives. The introduction, methods, results and discussion are well written and have a good theoretical basis, although I think the number of citations could be greatly reduced (more than 100 references used). Interesting results were found, such as a reduction in the time of calling activity and an earlier onset of the breeding period in urban bullfrog populations. In addition to carrying pathogens, bullfrogs may also represent acoustic overlap (niche) with native anuran species, as demonstrated by Medeiros et al. (2017 - doi: 10.1007/s10530-016-1327-7). All these findings may help to manage and control the populations of this invasive species. Therefore, this study is interesting for researchers who study the impacts of urbanization on biodiversity, for the ecology of invasive species and for herpetologists.
Still, some points require special attention. For example, regarding the use of the calling indices of P. nigromaculatus and D. japonicus. Nothing in the Introduction or in the study objectives provides support for evaluating the influence of the presence of other anuran species on the calling and reproduction activity of the American bullfrog.
In the attached file I have included some important suggestions and comments to improve the manuscript. Therefore, the study needs some minor revisions before being accepted for publication.

***Response:***

We highly appreciate your insightful comments and outlining the strength and significance of our manuscript. However, we have read your comments thoroughly and revised our manuscript accordingly. In addition, we have reviewed and reduced the number of references following your suggestion. Please check this rebuttal to explore the changes and corrections we made to our manuscript in response to your comments.

**Comment:**

Line 23-24: ‘This isn't a hypothesis. A hypothesis is an assumption, an idea that is proposed for the sake of argument so that it can be tested to see if it might be true. For example, in your study, a hypothesis would be something like: (i) frogs in urban environments will begin vocal activity earlier than those in non-urban areas; (ii) frog populations in urban areas will have shorter breeding seasons; (ii) urban noise will have negative effects on frog calling activity; and (iv) air and water temperature will positively influence frog calling activity.’

***Response:***

Thanks for your suggestion. We have deleted this sentence to avoid confusion,

**Comment:**

Line 25-26: ‘Suggestion: "which suggests a change induced by factors related to urbanization on reproduction phenology".’

***Response:***

We modified it in line 20-22.

**Comment:**

Line 29: ‘urban noise?’

***Response:***

We modified it in line 24.

**Comment:**

Line 30-31: ‘Ok, but what is the consequence of the results found?’

***Response:***

We appreciate your concern. We have added possible consequences of the results found. Please check line 25 – 27 of the revised version.

**Comment:**

Line 30: ‘frog breeding activities’

***Response:***

We modified it in line 28.

**Comment:**

Line 31-33: ‘How will this study help future research assess the impacts of urbanization on biodiversity and animal behavior?’

***Response:***

Thanks for your comment. We have deleted this sentence in the revised manuscript.

**Comment:**

Line 34: ‘This word is already in the title, change it to another one.’

***Response:***

Thanks for your concern. We have modified it in line 30 of the revised manuscript.

**Comment:**

Line 42-47: ‘Reference suggestion: Zaffaroni-Caorsi et al. 2022 - Effects of anthropogenic noise on anuran amphibians. https://doi.org/10.1080/09524622.2022.2070543’

***Response:***

We have included the suggested reference in the revised manuscript. Please check line 42.

**Comment:**

Line 98: ‘How will calling patterns be shaped? The response variable is the number of individuals in acoustic activity (Calling Index - CI), so the study is more related to breeding behavior (phenology).’

***Response:***

We modified it in line 92.

**Comment:**

Line 104: ‘Studied species and sites’

***Response:***

Thanks for your advice. We have modified it in the revised manuscript. Please check line 98.

**Comment:**

Line 110: ‘One reference is enough.’

***Response:***

We have modified it accordingly in line 104.

**Comment:**

Line 113: ‘Same comment as above.’

***Response:***

We have deleted additional references in line 107.

**Comment:**

Line 120: ‘Add a subtopic such as "Sampled Sites"’

***Response:***

We have added it in line 116.

**Comment:**

Line 127: ‘[63]’

***Response:***

We have deleted it in the revised manuscript.

**Comment:**

Line 164-166: ‘I saw in Table 3 that the call indexes (CI) of these species (Pelophylax nigromaculatus and Dryophytes japonicus) were included. However, I don't see much biological sense in adding these data to the model, since in the study hypothesis (not even mentioned in the introduction) there is nothing related to interspecific competition.’

***Response:***

Thanks for your comment. Both species are the most common sympatric species found with bullfrogs in South Korea, and they were included as just the sympatric species variables sharing habitat with bullfrog in the present study not to show the interspecific competition.

**Comment:**

Line 170: ‘What do you mean by "passing humans"?’

***Response:***

Thanks for your query. “Passing humans” means a person walking close to the recording equipment. However, we have modified the sentence to avoid confusions. Please check lines 165 – 166.

**Comment:**

Line 183-185: ‘Were human footsteps heard in the recordings? Do footsteps have a frequency higher than 90 Hz? This data seems to represent more the presence and absence of humans at that moment of the recording than "human noises".’

***Response:***

Thanks for your comment. Yes, we recorded it as presence and absence. Some sites (especially in type 3) had trails made of tree, and the sound of footsteps can be heard. The sounds were of more than 50Hz. As human noise was difficult to accurately estimate from a recorded file, we counted the presence and absence of human beings.

**Comment:**

Line 198: ‘ok, but I believe there may be some measurement error associated with this data, since it may be difficult to distinguish between two, three or four frogs calling at the same time, especially when they have similar call parameters and vocalize close to each other. Or not?’

***Response:***

We appreciate your comment. We agree with you that it is difficult to distinguish when many individuals call at the same time but not impossible. There are very small differences (dominant frequency, note duration, etc.) in the call of the individuals and we checked while watching spectrogram and listening to the sound source at the same time, so up to three frogs can be distinguished. You may also go through the following references for more information.

reference:
Zhang, F., Zhao, J., & Feng, A. S. (2017). Vocalizations of female frogs contain nonlinear characteristics and individual signatures. *PloS one*, *12*(3), e0174815. https://doi.org/10.1371/journal.pone.0174815
Pettitt, B. A., Bourne, G. R., & Bee, M. A. (2013). Advertisement call variation in the golden rocket frog (Anomaloglossus beebei): evidence for individual distinctiveness. *Ethology*, *119*(3), 244-256. https://doi.org/10.1111/eth.12058

**Comment:**

Line 243-244: ‘Add the average time of Type 3.’

***Response:***

We have added the average time of Type 3 in line 235.

**Comment:**

Line 260: ‘calling activity patterns’

***Response:***

We have modified it in line 25 of the revised manuscript.

**Comment:**

Line 267: ‘daily calling behavior’

***Response:***

We have modified it in line 258 – 259.

**Comment:**

Line 268-269: ‘This happens in South Korea, right? In tropical countries, the months (season) are quite different.’

***Response:***

Yes, this is the breeding season in South Korea. We have added the region to avoid confusion. Please check line 261.

**Comment:**

Line 315-317: ‘The mean energy concentration of trafic noise is below 1 kHz (Cunnington & Fahrig, 2010; Warren et al., 2006).

Cunnington, G. M., & Fahrig, L. (2010). Plasticity in the vocalizations of anurans in response to traffic noise. Acta Oecologica, 36, 463–470. https://doi.org/10.1016/j.actao.2010.06.002

Warren, P. S., Katti, M., Ermann, M., & Brazel, A. (2006). Urban bioacoustics: It's not just noise. Animal Behaviour, 71, 491–502. https://doi.org/10.1016/j.anbehav.2005.07.014’

***Response:***

Thanks for your comment. We read your reference articles carefully and thoroughly. We found 73.2 dBA traffic noise at ‘HIGH’ sites in the first reference (Cunnington and Fahrig, 2010), whereas second reference stated 74 dB at the airport flight paths and 70 dB at the highway (Warren et al., 2006). The conversion of the mentioned frequencies of the traffic noises in the reference articles give much higher reading than 1kHz. However, we also mentioned the references in the manuscript that support our statement that the traffic noise frequency can mask the Bullfrog call frequencies. We agree with you that the traffic noise and the Bullfrog call frequencies do not exactly match but can be partially overlapped. We plan to explore more on this issue that how and what exact influences of this partial overlapping will be on the frog calling behavior.

**Comment:**

Line 337-340: ‘It is important to highlight that not only temperature is associated with the beginning of acoustic activity in amphibians. Many species also require high humidity, which is higher at night when temperatures drop. Furthermore, at night amphibians avoid visually oriented predators (such as birds).’

***Response:***

We highly appreciate your insightful comment. We agree with you that the temperature is not the only factor for the onset of the frog calling behavior, and hence, we modified our statement accordingly in the revised version of the manuscript. Please check lines 323 – 325.

**Comment:**

Line 359: ‘in South Corea’

***Response:***

We have modified it in line 345.

**Comments from Reviewer #2:**

**Comment:**

Reviewer #2: The proposed article addresses a topic of great ecological interest, as it deals with the study of an invasive species of extreme importance. Furthermore, through the study of these populations, the authors shed light on the problem that the process of urbanization poses to these animal populations, and their results can be used as a tool to help understand and also to propose mitigating measures when this issue is involved. The study as a whole was very well conducted, and the methods used to obtain the results were also very well applied. I would like to make a small remark regarding the discussions related to your main finding, which is the influence of noise on the behavior of the studied anurans (for more details, see the body of the text), as well as other corrections/suggestions made throughout the text. I am available for any clarifications.

***Response:***

We highly appreciate your insightful comments and outlining the strength and significance of our manuscript. However, we have read your comments thoroughly and revised our manuscript accordingly.

**Comment:**

Line 1-3: ‘"I find it unnecessary for the authors to include the most important result of the work in the title... I suggest, just suggest, that the title be changed to 'The influence of urbanization on the breeding behavior of the American bullfrog (Aquarana catesbeiana) in areas with different levels of urbanization in South Korea'."’

***Response:***

Thanks for your suggestion. We have modified the title accordingly. Please check lines 1 – 2 of the revised manuscript.

**Comment:**

Line 14: ‘This statement cannot be generalized. There are urbanized areas that do not exhibit high levels of anthropogenic noise. Change this text to: "Urbanized areas often exhibit high levels of anthropogenic noise, which can mask or interfere with animal communication signals..."’

***Response:***

We have modified it in line 12.

**Comment:**

Line 37: ‘I insist on this condition of "urbanization"... small cities, villages, or places with urban infrastructure (such as schools, shops, and public services), where there is not a clear "high concentration of people", can be technically classified as urban areas, even if they do not have the same density or characteristics as large metropolitan areas. The category of "urban" encompasses a spectrum that includes both large cities and small localities. I suggest that the authors make this "adjustment/correction".’

***Response:***

We highly appreciate your suggestion. This definition was incorporated from the references cited with this sentence. We agree with you that small cities and villages with lower concentration of people in many cases contain urban infrastructure but are often indicated as sub-urban or developing areas. However, we have modified the sentence following your suggestion. Please check line 33 of the revised version.

**Comment:**

Line 42-43: ‘ok’

***Response:***

Thanks for your comment.

**Comment:**

Line 47-48: ‘Considering that a species is a concept, something human-designed as a hypothesis that reflects the evolutionary history of a group of individuals, you can never assert that "some species developed strategies..." or "some species change their behavior...". Just as we access some individuals of a population and not the species itself, you should phrase it as "individuals of this species developed strategies..." and so on.’

***Response:***

We have modified it in line 42.

**Comment:**

Line 49: ‘individuals of the Mientien tree frog...’

***Response:***

We have modified it in line 44.

**Comment:**

Line 50: ‘(Kurixalus idiootocus (Kuramoto & Wang, 1987)) calls...’

***Response:***

We have modified it in line 45.

**Comment:**

Line 61: ‘...that individuals of many species...’

***Response:***

We have modified it in line 55 – 56.

**Comment:**

Line 62: ‘For example, individuals of Alloopa hazarensis (Dubois & Khan, 1979)...

Correct the name of the genus (Allopaa).’

***Response:***

We have modified it in line 57 – 58.

**Comment:**

Line 63: ‘Nanorana vicina (Stoliczka, 1872)’

***Response:***

We have modified it in line 58.

**Comment:**

Line 72: ‘I don’t believe this is “a choice.” Issues like “choice” or “preferences” are very complex when it comes to nature... Individuals actually end up adapting, meaning they change their behavior to vocalize at “more suitable” times... Again, in my opinion, this is not a choice but a consequence...’

***Response:***

Thanks for your insightful comment. We have modified the sentence accordingly. Pease check line 66 – 69.

**Comment:**

Line 76: ‘It is not "creation", but "emergence of new habitats as a consequence of urbanization"...’

***Response:***

We have modified it in line 71.

**Comment:**

Line 79: ‘(Aquarana catesbeiana (Shaw, 1802)),’

***Response:***

We have modified it in line 74.

**Comment:**

Line 79-80: ‘Change the text to: "which was introduced into more than 40 nations on four continents and is now recognized as one of the most impactful invasive amphibian species in the world."’

***Response:***

Thanks for your suggestion. We have applied the changes in lines 74 – 76.

**Comment:**

Line 94: ‘... we studied the vocalization patterns of A.catesbeiana individuals and the number of calls over time’

***Response:***

We have modified it in lines 89 – 90.

**Comment:**

Line 105: ‘previously Rana (Lithobates) catesbeiana

see Fouquette, M. J., Jr., and A. Dubois. 2014. A Checklist of North American Amphibians and Reptiles. Seventh Edition. Volume 1—Amphibians. Bloomington, Indiana, USA: XLibris (Print-on-demand).’

***Response:***

We have modified it in lines 1000 – 101 following your suggestion.

**Comment:**

Line 165-166: ‘Pelophylax nigromaculatus (Hallowell, 1861)’

***Response:***

We have modified it in lines 162 – 163.

**Comment:**

Line 166: ‘Dryophytes japonicus (Günther, 1859)’

***Response:***

We have modified it in line 163.

**Comment:**

Line 199: ‘This information has already been provided in line 172. I suggest removing it.’

***Response:***

We highly appreciate your advice. We have deleted it in the revised manuscript.

**Comment:**

Line 209: ‘Furthermore, we conducted Watson-Williams F tests to compare the means, and Mardia tests to compare the significant distribution differences between two groups.’

***Response:***

We have modified it in lines 204 – 205.

**Comment:**

Line 215-222: ‘"We conducted the GLMMs using a Multinomial Logistic Regression because the CI of the bullfrog, which is a dependent variable, is a discrete variable ranging from 0 to 4. Except for the site, which we defined as a random effect, we defined all other variables as fixed effects. Since there was no significant correlation (r > 0.8) and no multicollinearity between the 14 variables, all variables were included in the analysis. We created 19 models with a combination of 14 variables. We used Akaike’s Information Criterion (AIC) to select the optimal model. We analyzed the AIC and performed the GLMMs using SPSS Statistics 23..."’

***Response:***

We have modified it in lines 208 – 216.

**Comment:**

Line 326-327: ‘You report a decrease in calling activity in areas with high noise levels and water temperatures (WT) between 16 and 27°C. OK! The discussion you present here regarding temperature is somewhat confusing: does it refer to air temperature (AT) or water temperature (WT), given that you do not provide any discussion about air temperature? I understand that water temperature is also related to air temperature, but at no point do you report the values for air temperature... notice that the discussion based on Kaefer et al. (2007) is solely about air temperature, and Milne et al. (2013) considers both air and water temperatures ("However, gray tree frogs were temperature sensitive and calling was significantly related to increased water and air temperatures")... This part is really confusing! I recognize that water temperature is fundamental to the behavior of individuals with an aquatic habit and that, again, there is a relationship between air and water temperatures, but these issues need to be very clear when you discuss these two variables in this section.’

***Response:***

Thanks for your comment. We agree with you. We have modified this part in the revised version to avoid the confusion. Please check lines 316 – 319.

**Comment:**

Line 338-339: ‘As I mentioned above, regarding air temperature (AT): "...though A. catesbeiana depends on the seasonal temperature, it begins calling only when the water temperature reaches a suitable range"... "showed higher temperatures than the other types, and thus, this may have influenced early breeding phenology and calling activities." - I ask: is it air temperature or water temperature? Again: I know that the temperatures are related, but they are not the same. So, it's important to clarify which temperature you are referring to (AT or WT)?’

***Response:***

We highly appreciate your concern. As you mentioned that the temperatures are interrelated, we meant both AT and WT. However, we have modified the sentence and specified the water temperature in the revised manuscript. Please check lines 326 – 328.

**Comment:**

Line 345-346: ‘Note that the temperature referred to by these authors is air temperature (AT)...’

***Response:***

Thanks for your comment. We have added reference for WT in the revised version. Please check line 335.

**Comment:**

Line 348-349: ‘The influence of environmental temperature (or water temperature) on the behavior of these animals is somewhat obvious, considering that we are dealing with amphibians. But what is the real importance of noise/urbanization in this process? This is indeed an important point for discussion, given the reality of current environments in most habitats occupied by A. catesbeiana , as well as many other amphibian species. You observed only 34 calls in the presence of continuous traffic noise, while in the absence of this noise, the number of calls reached 7,430. This result is very telling! It is clear that the variable 'noise' is much more important here than water or air temperature. (It would be different if you were using a modeling tool to predict how this species would adapt or not under future climate change conditions and the consequent alteration of environmental (and water) temperature... but that is not the issue at hand.) I suggest exploring this question (noise/urbanization) further, as it is, as stated in the title of your study, the central problem to be investigated in your research!’

***Response:***

Thanks for your insightful comment and recognition of the significance of our study. We agree with you that it would have been better if we could incorporate a modeling tool to predict how this species would adapt or not under future climate change conditions and the consequent alteration of environmental (and water) temperature. But we exclude future climate conditions to be more focused on the present condition in the field and stick to the primary goal of the study. For the discussion on the question about the real importance of noise/urbanization in this process, we request you read the first paragraph of section ‘4.3 Factors influencing bullfrog calling activity’ under ‘4. Discussion’ again. We have already discussed this issue there elaborately. However, we also admit that the extent of this study and the dimensions of the discussion of this issue can be further expanded. Thus, we have suggested further extensive studies and exploration of the related real consequences of noise/urbanization on breeding behavior in amphibians following your advice. We also have a plan to extend this study to the next level, where we expect to incorporate all your suggestions that we might have somehow missed in the current manuscript.
